# A conformational switch in initiation factor 2 controls the fidelity of translation initiation in bacteria

**DOI:** 10.1038/s41467-017-01492-6

**Published:** 2017-11-14

**Authors:** Kelvin Caban, Michael Pavlov, Måns Ehrenberg, Ruben L. Gonzalez

**Affiliations:** 1 Department of Chemistry, Columbia University, 3000 Broadway, MC3126, New York, NY 10027 USA; 2 Department of Cell and Molecular Biology, BMC, Uppsala University, Husargatan 3, Uppsala, 751 24 Sweden

## Abstract

Initiation factor (IF) 2 controls the fidelity of translation initiation by selectively increasing the rate of 50S ribosomal subunit joining to 30S initiation complexes (ICs) that carry an *N*-formyl-methionyl-tRNA (fMet-tRNA^fMet^). Previous studies suggest that rapid 50S subunit joining involves a GTP- and fMet-tRNA^fMet^-dependent “activation” of IF2, but a lack of data on the structure and conformational dynamics of 30S IC-bound IF2 has precluded a mechanistic understanding of this process. Here, using an IF2-tRNA single-molecule fluorescence resonance energy transfer signal, we directly observe the conformational switch that is associated with IF2 activation within 30S ICs that lack IF3. Based on these results, we propose a model of IF2 activation that reveals how GTP, fMet-tRNA^fMet^, and specific structural elements of IF2 drive and regulate this conformational switch. Notably, we find that domain III of IF2 plays a pivotal, allosteric, role in IF2 activation, suggesting that this domain can be targeted for the development of novel antibiotics.

## Abstract (web summary)

The GTP-bound form of initiation factor 2 (IF2) promotes translation initiation by accelerating 50S ribosomal subunit joining the 30S ribosomal initiation complex (30S IC). Here the authors use single-molecule FRET and ensemble rapid kinetic methods to uncover the mechanism behind IF2-mediated subunit joining.

## Introduction

Initiation of bacterial protein synthesis, or translation, proceeds along a multi-step pathway that begins with the assembly of a 30S initiation complex (IC) (Supplementary Fig. 1a). The 30S IC is composed of the small (30S) ribosomal subunit, initiation factor (IF) 1, the guanosine triphosphatase (GTPase) IF2, IF3, initiator *N*-formyl-methionyl-transfer RNA (fMet-tRNA^fMet^), and messenger RNA (mRNA). Although 30S IC assembly can occur via multiple pathways^1^, a kinetically favored pathway has been identified in which the three IFs bind to the 30S subunit and synergistically regulate the kinetics of tRNA binding^2^. Consequently, fMet-tRNA^fMet^ is preferentially selected into the peptidyl-tRNA-binding (P) site of the 30S subunit, where it base-pairs to the start codon of an mRNA that can bind to the 30S subunit before, during, or after the IFs bind^2–7^. The IFs further enhance the accuracy of translation by cooperatively regulating the rate with which the large (50S) ribosomal subunit joins to the 30S IC and by modulating the stability of the resulting 70S IC^4,5,7–11^. 70S IC formation triggers GTP hydrolysis by IF2, which subsequently drives a series of maturation steps that enable the 70S IC to enter the elongation stage of protein synthesis^12–14^.

IF2 plays central roles throughout the initiation pathway that ensure accurate fMet-tRNA^fMet^ selection. During 30S IC assembly, IF2 specifies fMet-tRNA^fMet^ selection by interacting with the *N*-formyl-methionine and aminoacyl acceptor stem of fMet-tRNA^fMet^ ^15–18^. IF2 further ensures the accuracy of fMet-tRNA^fMet^ selection by preferentially accelerating the rate with which the 50S subunit joins to a 30S IC carrying fMet-tRNA^fMet^ ^4,5,9,19–21^. Indeed, 50S subunit joining to 30S ICs carrying GTP-bound IF2 (IF2(GTP)) and P site-bound fMet-tRNA^fMet^ is up to between one and two orders of magnitude faster than to 30S ICs in which GTP has been substituted with GDP or to “pseudo” 30S ICs in which fMet-tRNA^fMet^ has been substituted with an unformylated Met-tRNA^fMet^, unacylated tRNA^fMet^, elongator tRNA, or no tRNA at all^4,5,19–21^.

IF2 consists of four conserved structural domains, referred to here as domains I-IV (dI-IV) in the nomenclature of Roll-Mecak et al.^22^, but also referred to as domains G2 (dI), G3 (dII), C1 (dIII), and C2 (dIV) in the nomenclature of Gualerzi et al.^23^ or as domains dIV (dI), dV (dII), dVI-1 (dIII), and dVI-2 (dIV) in the nomenclature of Mortensen et al.^24^ The arrangement of these domains is such that dII and dIII separate the guanine nucleotide-binding domain, dI, from the fMet-tRNA^fMet^-binding domain, dIV. Structural studies of non-ribosome-associated IF2 strongly suggest that the spatial positions of dIII and dIV are flexible relative to dI and dII, allowing IF2 to adopt increasingly extended conformations upon transitions from nucleotide-free IF2 to IF2(GDP) and IF2(GTP)^25,26^. Within the context of the 30S IC, dII helps anchor IF2(GTP) to the 30S IC by interacting with 16S ribosomal RNA (rRNA) helices h5 and h14^16^. Moreover, dIII and dIV adopt positions relative to dI and dII that enable dIV to interact with the P site-bound fMet-tRNA^fMet^ ^16^. These interactions, which might be further stabilized by the interactions of dIII with ribosomal protein S12^27^, result in the formation of an IF2(GTP)•tRNA sub-complex on the inter-subunit surface of the 30S IC^16–18^.

Previously, Andersson and colleagues^20^ identified IF2 variants containing single amino acid substitution mutations within dIII (mutIF2s) that, remarkably, enable mutIF2(GDP)s to catalyze rapid 50S subunit joining to 30S ICs and mutIF2(GTP)s to catalyze rapid 50S subunit joining to pseudo 30S ICs^20,21^. Based on these results, we have proposed that IF2 is “activated” for rapid 50S subunit joining by a GTP- and fMet-tRNA^fMet^-dependent conformational switch that is rendered GTP- and fMet-tRNA^fMet^-independent by the “activating” mutations in dIII of the mutIF2s^20,21^. Nonetheless, due to a lack of experimental data on the structure of IF2(GDP)-bound 30S ICs, IF2(GTP)-bound pseudo 30S ICs, mutIF2(GDP)-bound 30S ICs, and/or mutIF2(GTP)-bound pseudo 30S ICs, as well as on the GTP- and fMet-tRNA^fMet^-dependent conformational dynamics of 30S IC-bound IF2 and mutIF2, the structural basis and molecular mechanism of IF2 activation have remained unknown.

To close this gap in our understanding of how IF2 helps regulate the fidelity of translation initiation, here we report an investigation of the structural dynamics of GTP- and fMet-tRNA^fMet^-dependent IF2 activation using single-molecule fluorescence resonance energy transfer (smFRET). Our data provide direct evidence that IF2 activation consists of a conformational switch of IF2 and demonstrate that the GTP- and fMet-tRNA^fMet^-dependent dynamics of this switch regulates IF2 activation by modulating the affinity of IF2 for the 30S IC and the conformation of 30S IC-bound IF2. Based on these results, we propose a model for IF2 activation specifying how GTP, fMet-tRNA^fMet^, and the four domains of IF2 collectively drive and regulate the dynamics of this conformational switch. Interestingly, we find that dIII allosterically regulates IF2 activation, highlighting dIII as an attractive target for the development of novel antibiotics that function as allosteric inhibitors of IF2.

## Results

### *Escherichia coli* mutIF2 catalyzes rapid 50S subunit joining

mutIF2s were initially selected in *Salmonella (S) typhimurium* on the basis of their ability to complement the slow growth phenotype arising from a Met-tRNA^fMet^ formylation deficiency^20^. One such *S. typhimurium* mutIF2 contains a Ser755Tyr mutation in dIII and has been shown to strongly compensate for a Met-tRNA^fMet^ formylation deficiency both in vivo and in vitro^20,21^. Here, we generated the homologous *E. coli* Ser753Tyr mutIF2 (Supplementary Fig. 2), purified it, and confirmed its ability to catalyze rapid 50S subunit joining to both 30S ICs and pseudo 30S ICs using ensemble kinetic studies of subunit joining (Supplementary Fig. 3). Importantly, a Gly810Cys mutation in dIV, previously used to label *E. coli* IF2 with a FRET acceptor fluorophore (ref. ^28^ and vide infra), did not alter the kinetic performance of either *E. coli* IF2(GTP) or *E. coli* Ser753Tyr mutIF2(GTP). We further validated the biochemical activities of our unlabeled IF2 variants using a standard, biochemical IF2 activity assay that is based on primer extension inhibition, or “toeprinting” (Supplementary Fig. 4). Unless otherwise specified, the designations “wtIF2” and “mutIF2” will hereafter refer to *E. coli* wild-type IF2 and *E. coli* Ser753Tyr mutIF2, respectively, both harboring an additional Gly810Cys mutation in dIV.

### wtIF2(GTP) and mutIF2(GTP) adopt similar conformations

To characterize the interaction of wtIF2 and mutIF2 with 30S ICs and pseudo 30S ICs, we used a previously developed IF2-tRNA smFRET signal^28^. This signal reports on changes in the distance between a cyanine 5 (Cy5) FRET acceptor fluorophore in dIV of IF2 (wtIF2[Cy5]_dIV_ or mutIF2[Cy5]_dIV_) and a cyanine 3 (Cy3) FRET donor fluorophore in the central fold, or “elbow”, domain of tRNA^fMet^ (tRNA(Cy3)^fMet^), thereby reporting on the formation and conformational dynamics of the IF2•tRNA sub-complex (Supplementary Fig. 1b). We began by assembling a 30S IC using 30S subunits, a 5′-biotinylated mRNA, fMet-tRNA(Cy3)^fMet^, IF1, wtIF2[Cy5]_dIV_, and GTP (hereafter referred to as 30S IC_wT_, where the “w” and “T” subscripts denote wtIF2[Cy5]_dIV_ and GTP, respectively). Previously, we have shown that IF3 destabilizes the binding of all tRNAs to the 30S subunit P site^4,5,29^; thus, IF3 was excluded from the assembly of all of the 30S ICs and pseudo 30S ICs in the current study. We note that, even in the absence of IF3, IF2 retains the ability to selectively accelerate the rate of 50S subunit joining to correctly assembled 30S ICs^4,5,21^. Furthermore, exclusion of IF3 provides a simple model system to allow for clarification of the basal conformational changes of 30S IC-bound IF2 that confer rapid and selective 50S subunit joining. Following previously published protocols^28^, 30S IC_wT_ was then tethered to the surface of a quartz microfluidic flowcell and imaged at single-molecule resolution using a total internal reflection fluorescence (TIRF) microscope operating at an acquisition time of 0.1 s per frame. As before^28^, we supplemented all buffers with 25 nM wtIF2[Cy5]_dIV_(GTP) in order to allow re-association of wtIF2[Cy5]_dIV_(GTP) with 30S IC_wT_s from which it might have dissociated during tethering and/or TIRF imaging.

**Fig. 1:**
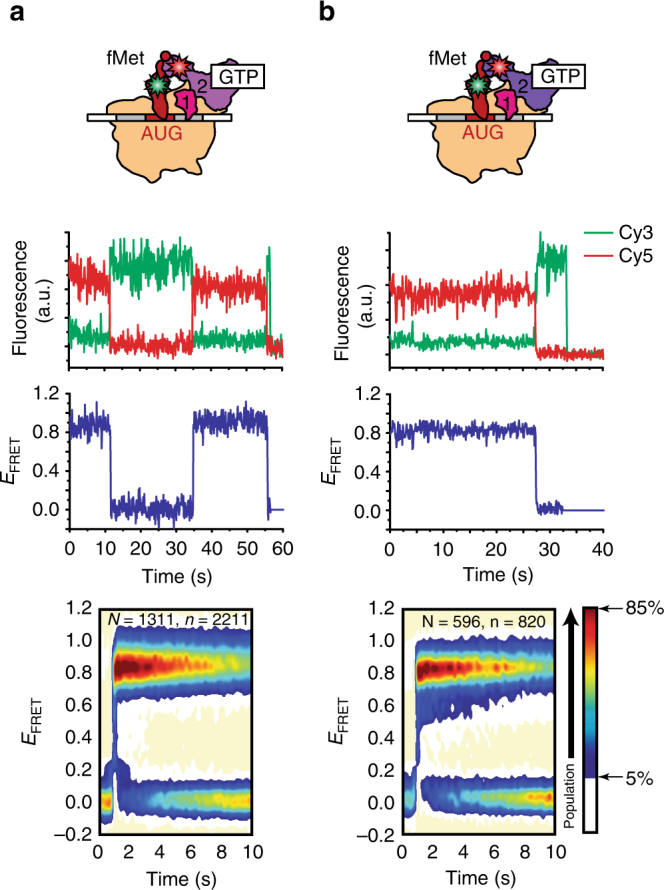
Effect of GTP and fMet-tRNA^fMet^. smFRET measurements of (**a**) wtIF2(GTP) and (**b**) mutIF2(GTP) interacting with 30S IC_wT_ and 30S IC_mT_, respectively. First row: cartoon illustrations depicting 30S IC_wT_-bound wtIF2(GTP) (light purple) and 30S IC_mT_-bound mutIF2(GTP) (dark purple). Second row: representative Cy3 (green) and Cy5 (red) emission intensities vs. time trajectories. Third row: corresponding *E*_FRET_ vs. time trajectories. Fifth row: post-synchronized surface contour plots of the time evolution of population FRET. Surface contour plots were generated by superimposing hundreds of individual IF2-binding events. “*N*” indicates the total number of *E*_FRET_ vs. time trajectories for each 30S IC and “*n*” indicates the total number of individual IF2-binding events. The surface contours were plotted from tan (lowest population plotted) to red (highest population plotted) as indicated in the population color bar

Consistent with our previous smFRET studies^28^, individual FRET efficiency (*E*_FRET_) vs. time trajectories exhibited reversible fluctuations between a zero FRET state, corresponding to the IF2-free state of 30S IC_wT_, and a non-zero FRET state, corresponding to the wtIF2(GTP)-bound state of 30S IC_wT_ (Fig. 1a). Kinetic and thermodynamic parameters describing the interaction of wtIF2(GTP) with 30S IC_wT_ were determined using previously described methods (see ref. ^28^ and Methods section). Briefly, we learned a hidden Markov model (HMM) from the *E*_FRET_ trajectories to determine the probabilities of transitioning between the IF2-free- and wtIF2(GTP)-bound states of 30S IC_wT_ and converted the resulting state transition probabilities into rate constants using a transition probability matrix-based population decay analysis. Using this approach, we determined the bimolecular rate constant for the association of wtIF2(GTP) to 30S IC_wT_ (*k*_a,wT_) to be 2.0 ± 0.1 μM^−1^ s^−1^, the rate constant for the dissociation of wtIF2(GTP) from 30S IC_wT_ (*k*_d,wT_) to be 0.041 ± 0.01 s^−1^, and the equilibrium dissociation constant for the wtIF2(GTP)–30S IC_wT_ complex (*K*_d,wT_) to be 21 ± 6 nM (Table 1).

**Table 1:** Association rate constant (*k*_a_), dissociation rate constant (*k*_d_), and dissociation equilibrium constant (*K*_d_) for the interaction of IF2 and the 30S IC

30S IC	IF2	Nucleotide	*k*_a_ (μM^–1^ s^–1^)^a^	*k*_d_ (s^–1^)^a^	*K*_d_ (nM)^a^
30S IC_wT_	wtIF2	GTP	2.0 ± 0.13^b^	0.041 ± 0.01^b^	21 ± 6
30S IC_mT_	mutIF2	GTP	2.2 ± 0.4^b^	0.013 ± 0.001^b^	6.4 ± 1.3
30S IC_wD_	wtIF2	GDP	2.1 ± 0.12	1.32 ± 0.09	622 ± 28
30S IC_mD_	mutIF2	GDP	1.2 ± 0.10	0.13 ± 0.01	102 ± 9
30S IC_wT,Met_	wtIF2	GTP	0.52 ± 0.02^c^	1.2 ± 0.2	2328 ± 294
30S IC_mT,Met_	mutIF2	GTP	0.77 ± 0.05	0.11 ± 0.01	136 ± 7
30S IC_wT,OH_	wtIF2	GTP	0.38 ± 0.02^c^	2.2 ± 0.5	5842 ± 1469
30S IC_mT,OH_	mutIF2	GTP	0.74 ± 0.02	0.14 ± 0.02	185 ± 18

^a^ *k*_a_, *k*_d_, and *K*_d_ were obtained from three independently collected data sets (mean ± SE) using a transition probability matrix-based population decay analysis as described previously^28^ and in Methods section

^b^ *k*_a_ and *k*_d_ were corrected for the effects of Cy5 photobleaching

^c^ *k*_a_ was corrected for the effects of Cy3 photobleaching

To characterize the conformational dynamics of the wtIF2(GTP) tRNA sub-complex on 30S IC_wT_, we plotted histograms of the *E*_FRET_ values observed for the wtIF2(GTP)-bound state of 30S IC_wT_ (Fig. 1a and Supplementary Fig. 5a). The distribution of *E*_FRET_ values exhibited a single non-zero *E*_FRET_ peak that was centered at a mean *E*_FRET_ value (<*E*_FRET_>) of 0.87 ± 0.02 (Supplementary Table 1). Using a Förster distance (*R*_0_) of 55 Å for the Cy3-Cy5 FRET donor–acceptor pair^30^ and assuming unrestricted, isotropic motion of the fluorophores, this <*E*_FRET_> corresponds to an ~40 Å average separation between our labeling positions, a separation that is consistent with the cryogenic electron microscopy (cryo-EM) structure of 30S IC-bound IF2(GTP)^18^.

To investigate the effects that the activating mutation in dIII has on the affinity of IF2(GTP) for the 30S IC and the conformation of 30S IC-bound IF2(GTP), we performed smFRET experiments using mutIF2[Cy5]_dIV_(GTP) and 30S IC_mT_ (where the “m” subscript denotes mutIF2[Cy5]_dIV_). The results demonstrate that excursions to the mutIF2(GTP)-bound state in the 30S IC_mT_ *E*_FRET_ trajectories are longer-lived than those to the wtIF2(GTP)-bound state in the 30S IC_wT_ *E*_FRET_ trajectories (compare Fig. 1a and b). Consistent with this, *K*_d,mT_ is approximately threefold smaller than *K*_d,wT_ (Table 1), demonstrating that the activating mutation confers a higher affinity of mutIF2(GTP) for 30S IC_mT_ than the affinity of wtIF2(GTP) for 30S IC_wT_. Interestingly, the distribution of *E*_FRET_ values for the mutIF2(GTP)-bound state of 30S IC_mT_ (Fig. 1b and Supplementary Fig. 5b) was composed of a single non-zero *E*_FRET_ peak that was centered at an <*E*_FRET_> of 0.85 ± 0.01 that is within error of that observed for the wtIF2(GTP)-bound state of 30S IC_wT_ (*p* value = 0.2, Supplementary Table 1). This indicates that the conformation of 30S IC_mT_-bound mutIF2(GTP) is not significantly altered by the activating mutation and is very similar to that of a 30S IC_wT_-bound wtIF2(GTP). Previously, we have used ensemble kinetic experiments to show that wtIF2(GTP) and mutIF2(GTP) can catalyze rapid 50S subunit joining to 30S IC_wT_* and 30S IC_mT_* (where the asterisk denotes the analogous 30S IC in the kinetic studies)^4,5,20,21^. We therefore interpret the observed <*E*_FRET_> s of ~0.85 and ~0.87 as corresponding to a conformation of the IF2(GTP)•tRNA sub-complex in which IF2(GTP) is active for rapid 50S subunit joining.

### GTP allosterically positions dIV closer to the P-site tRNA

**Fig. 2:**
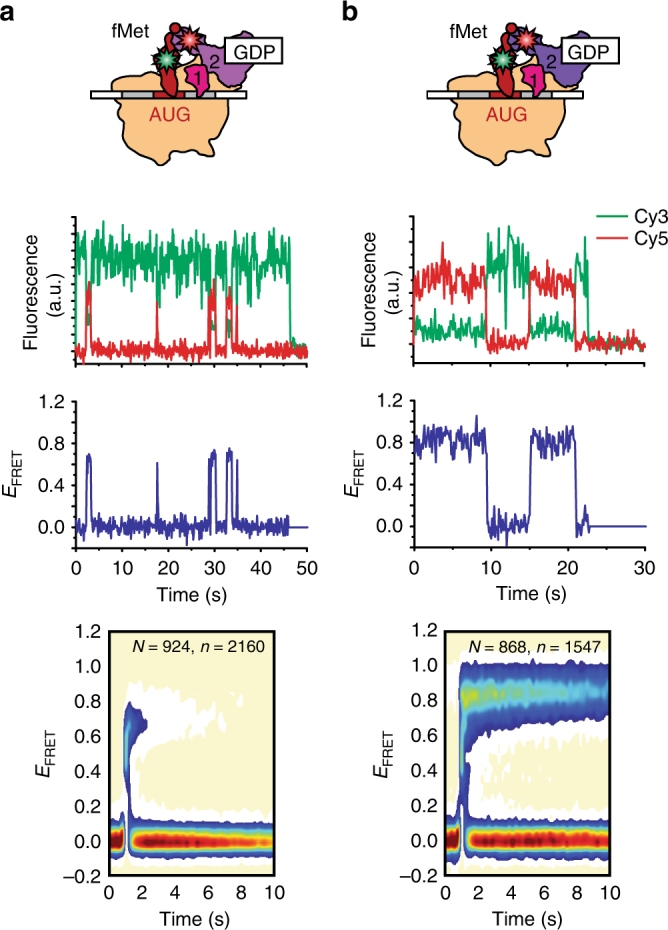
Effect of substituting GTP with GDP. smFRET measurements of (**a**) wtIF2(GDP) and (**b**) mutIF2(GDP) interacting with 30S IC_wD_ and 30S IC_mD_, respectively. Data are displayed as in Fig. 1

We next performed smFRET experiments using wtIF2[Cy5]_dIV_(GDP) and 30S IC_wD_ (where the “D” subscript denotes GDP) to explore if and how the affinity of IF2 for the 30S IC and the conformation of 30S IC-bound IF2 depend on the guanine nucleotide that is bound to IF2. These experiments reveal that excursions to the wtIF2(GDP)-bound state in the 30S IC_wD_ *E*_FRET_ trajectories are more transient than those to the wtIF2(GTP)-bound state in the 30S IC_wT_ *E*_FRET_ trajectories (compare Figs. 2a and 1a). Correspondingly, we observe a *K*_d,wD_ value that is ~30-fold larger than the *K*_d,wT_ value (Table 1), demonstrating that the affinity of wtIF2 binding to the 30S IC is much higher when GTP, rather than GDP, is bound to IF2. In addition, we found that the distribution of *E*_FRET_ values for the wtIF2(GDP)-bound state of 30S IC_wD_ (Fig. 2a and Supplementary Fig. 5c) exhibited two non-zero *E*_FRET_ peaks. One of the peaks encompassed a minor, 18 ± 1.5%, subpopulation of 30S IC_wD_-bound wtIF2(GDP) and was centered at an <*E*_FRET_> of 0.89 ± 0.01 that is within error of that observed for 30S IC_wT_-bound wtIF2(GTP) (*p* value = 0.2, Supplementary Table 1). The other peak encompassed a major, 82 ± 1.5%, subpopulation of 30S IC_wD_-bound wtIF2(GDP) and was centered at an <*E*_FRET_> of 0.67 ± 0.01 that is notably lower than that observed for 30S IC_wT_-bound wtIF2(GTP) (*p* value = 0.002, Supplementary Table 1).

Previously, we have used ensemble kinetic experiments to show that 30S IC_wD_* exhibits a drastic, ~60-fold smaller rate of 50S subunit joining than 30S IC_wT_*^21^. Based on the values of *K*_d,wD_ and *K*_d,wT_ determined here (622 nM and 21 nM, respectively) and the IF2 and 30S IC concentrations employed in our previous kinetic studies of 50S subunit joining^21^, we estimate that the occupancy of wtIF2(GDP) on 30S IC_wD_* in our previous studies was only twofold lower than the occupancy of wtIF2(GTP) on 30S IC_wT_* (Supplementary Table 2). Thus, this occupancy difference is insufficient to account for the decreased rate of 50S subunit joining to 30S IC_wD_*. Instead, we conclude that the decreased rate of 50S subunit joining primarily arises from the stabilization of a major subpopulation of 30S IC_wD_-bound wtIF2(GDP) in a conformation that is inactive for rapid 50S subunit joining and that, given our measured <*E*_FRET_> values, features a separation between our labeling positions that is ~9 Å longer than what it is in a 30S IC_wT_-bound wtIF2(GTP) that is active for rapid 50S subunit joining. Given that dIV is connected to dIII via a potentially flexible linker^34^, this ~9 Å increase in the distance between dIV and the P-site tRNA can arise from two different scenarios. In the first scenario, dIV adopts a single, fixed position that is ~49 Å from the P-site tRNA. In the alternative scenario, dIV adopts multiple positions that interconvert on a timescale that is faster than the acquisition time of our TIRF microscope (i.e., 0.1 s per frame), yielding a time-averaged position that is ~49 Å from the P-site tRNA.

Such a difference between the conformations of the GDP- and GTP-bound forms of IF2 is consistent with comparative structural analyses of non-ribosome-associated IF2(GDP) and IF2(GTP)^26^ and of 70S IC-bound IF2(GDP)^31^ and IF2(GTP)^27,31–33^. Based on these analyses, we propose that the guanine nucleotide bound to dI of 30S IC-bound IF2 allosterically modulates the position of dIV relative to that of dI-dIII and the P-site tRNA. Indeed, compared to the position of dIV in IF2(GDP) in these structures, dIV in IF2(GTP) is positioned further away from dI-dIII and closer to the P-site tRNA.

To validate this model, we developed a wtIF2 variant in which dIII was labeled with a Cy5 fluorophore (wtIF2[Cy5]_dIII_), Methods section), and used wtIF2[Cy5]_dIII_ to repeat the smFRET experiments described above. We found that the distributions of *E*_FRET_ values for the wtIF2(GTP)-bound state of 30S IC_wT_ and the wtIF2(GDP)-bound state of 30S IC_wD_ were both composed of only a single non-zero *E*_FRET_ peak that was centered at an <*E*_FRET_> of ~0.3 and an average distance between our labeling positions of ~63 Å (Supplementary Fig. 6). These results strongly suggest that the relative distance between dIII and the P-site tRNA is similar in the minor and major subpopulations of 30S IC_wD_-bound wtIF2(GDP) and that this distance is comparable to the corresponding distance in 30S IC_wT_-bound wtIF2(GTP). Notably, however, we were able to unambiguously identify two kinetically distinguishable subpopulations of the wtIF2(GDP)-bound state of 30S IC_wD_ whose kinetic properties were equivalent to those of the minor and major subpopulations of the wtIF2(GDP)-bound state of 30S IC_wD_ that we identified using wtIF2[Cy5]_dIV_ (Supplementary Fig. 7). Collectively, the data obtained using wtIF2[Cy5]_dIII_ and wtIF2[Cy5]_dIV_ allows us to validate and extend the structural model described above.

### The activating mutation in dIII allosterically positions dIV

To determine whether and how the activating mutation in dIII modulates the affinity of IF2(GDP) for the 30S IC and the conformation of 30S IC-bound IF2(GDP), we performed smFRET experiments using mutIF2[Cy5]_dIV_(GDP) and 30S IC_mD_. The results show that excursions to the mutIF2(GDP)-bound state in the 30S IC_mD_ *E*_FRET_ trajectories are significantly longer than those to the wtIF2(GDP)-bound state in the 30S IC_wD_ *E*_FRET_ trajectories (compare Fig. 2a and b). In line with this, we find that the value of *K*_d,mD_ is approximately sixfold smaller than that of *K*_d,wD_ (Table 1). Thus, the activating mutation in dIII enables mutIF2(GDP) to bind to 30S IC_mT_ with a higher affinity than wtIF2(GDP) binds to 30S IC_wD_. More importantly, however, we find that the distribution of *E*_FRET_ values for the mutIF2(GDP)-bound state of 30S IC_mD_ (Fig. 2b and Supplementary Fig. 5d) is composed of a single non-zero *E*_FRET_ peak centered at an <*E*_FRET_> of 0.86 ± 0.03 that is within error of that observed for the wtIF2(GTP)-bound state of 30S IC_wT_ (*p* value = 0.8, Supplementary Table 1). Thus, remarkably, the activating mutation in dIII enables 30S IC_mD_-bound mutIF2(GDP) to adopt a conformation that closely resembles that observed for a 30S IC_wT_-bound wtIF2(GTP) that is active for rapid 50S subunit joining.

Previously, we have used ensemble kinetic experiments to show that the rate of 50S subunit joining to 30S IC_mD_* is ~40-fold higher than to 30S IC_wD_*^21^. Thus, the activating mutation in dIII enables mutIF2(GDP) to catalyze 50S subunit joining to 30S IC_mD_* at a rate similar to that observed for 50S subunit joining to 30S IC_wT_*. Based on the results reported here, we propose that the activating mutation in dIII enables rapid 50S subunit joining by stabilizing a conformation of dI-dIII that increases the affinity of mutIF2(GDP) for 30S IC_mD_ and that enables mutIF2(GDP) to position dIV closer to the P site such that it can interact with the P site-bound fMet-tRNA^fMet^. Moreover, our result also implies that stabilizing the analogous conformation of 30S IC-bound wtIF2 requires the binding of GTP to dI. Hence, it is the conformation of dI-dIII, which in wtIF2 is specified by the guanine nucleotide that is bound to dI, that determines the position of dIV relative to the P-site tRNA and controls the activation of 30S IC-bound IF2 for rapid 50S subunit joining.

### fMet-tRNA^fMet^ stabilizes the active conformation of wtIF2

**Fig. 3:**
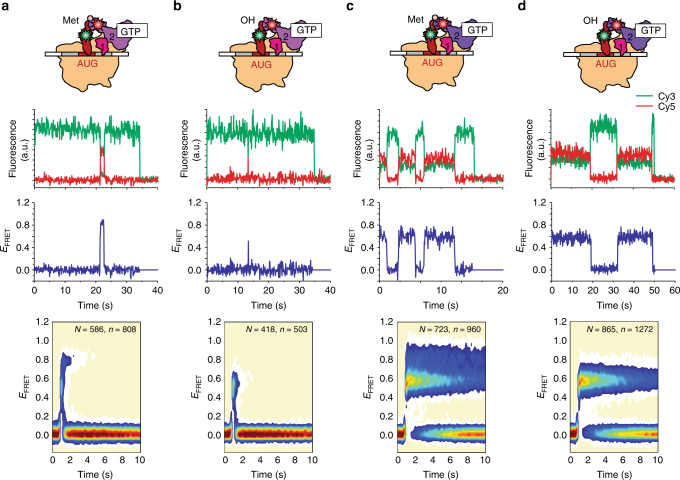
Effect of substituting fMet-tRNA^fMet^ with Met-tRNA^fMet^ or tRNA^fMet^. smFRET measurements of (**a**, **b**) wtIF2(GTP) and (**c**, **d**) mutIF2(GTP) interacting with (**a**, **c**) 30S IC_wT,Met_ or 30S IC_mT,Met_, respectively, and to (**b**, **d**) 30S IC_wT,OH_ or 30S IC_mT,OH_, respectively. Data are displayed as in Fig. 1

Next, we performed smFRET experiments using wtIF2[Cy5]_dIV_(GTP) and analogs of 30S IC_wT_ in which the fMet-tRNA^fMet^ has been substituted with Met-tRNA^fMet^ (30S IC_wT,Met_) or tRNA^fMet^ (30S IC_wT,OH_) to investigate if and how the affinity of IF2 for the 30S IC and the conformation of 30S IC-bound IF2 depend on the *N*-formyl moiety and/or methionine of the 30S IC-bound fMet-tRNA^fMet^. Consistent with our previous smFRET studies^28^, we found that the 30S IC_wT,Met_ and 30S IC_wT,OH_ *E*_FRET_ trajectories exhibit excursions to the wtIF2(GTP)-bound state that are much shorter lived than those of 30S IC_wT_ (compare Fig. 3a, b with Fig. 1a). In line with this, *K*_d,wT,Met_ is ~100-fold and *K*_d,wT,OH_ is ~300-fold larger than *K*_d,wT_ (Table 1). These results suggest that the absence of just the *N*-formyl moiety or the *N*-formyl-methionine from the 30S IC-bound fMet-tRNA^fMet^ is enough to disrupt interactions between dIV and fMet-tRNA^fMet^ that significantly contribute to anchoring wtIF2(GTP) to the 30S IC.

Consistent with our previous smFRET studies^28^, we find that the distribution of *E*_FRET_ values for the wtIF2(GTP)-bound state of 30S IC_wT,Met_ (Fig. 3a and Supplementary Fig. 5e) is very broad, with values in the 0.2–1.0 range that encompass two non-zero *E*_FRET_ peaks. The peak corresponding to the larger, 56 ± 12%, subpopulation of 30S IC_wT,Met_-bound wtIF2(GTP) was centered at an <*E*_FRET_> of 0.81 ± 0.01 that is outside the error of that observed for 30S IC_wT_-bound wtIF2(GTP) (*p* value = 0.08, Supplementary Table 1). This observation suggests that the separation between our labeling positions is ~43 Å in this subpopulation of 30S IC_wT_-bound wtIF2(GTP), a separation that is ~3 Å longer than what it is in a 30S IC_wT_-bound wtIF2(GTP) that is active for rapid 50S subunit joining. The peak corresponding to the smaller, 44 ± 12%, subpopulation of 30S IC_wT,Met_-bound wtIF2(GTP) was centered at an even lower <*E*_FRET_> of 0.55 ± 0.01, indicating that the distance between our labeling positions is ~53 Å, ~13 Å longer than what it is in 30S IC_wT_-bound wtIF2(GTP) that is active for rapid 50S subunit joining. Even more dramatic results are obtained for the distribution of *E*_FRET_ values for the wtIF2(GTP)-bound state of 30S IC_wT,OH_ (Fig. 3b and Supplementary Fig. 5g) in that the distribution exhibits only a single non-zero *E*_FRET_ peak that is centered at an <*E*_FRET_> of 0.53 ± 0.02 that is within error of that observed for the smaller subpopulation of 30S IC_wT,Met_-bound wtIF2(GTP) (*p* value = 0.4, Supplementary Table 1).

Our previous ensemble kinetic studies have shown that the rates of 50S subunit joining to 30S IC_wT,Met_* and 30S IC_wT,OH_* are approximately fourfold and ~15-fold lower, respectively, than that to 30S IC_wT_*^21^. Given the values of *K*_d,wT,Met_ and *K*_d,wT,OH_ determined here and of the wtIF2 and 30S IC_wT,Met_* and 30S IC_wT,OH_* concentrations used in our previous kinetic studies^21^, we estimate that the occupancy of wtIF2(GTP) on 30S IC_wT,Met_* and 30S IC_wT,OH_* in our previous kinetic studies was approximately fivefold and ~10-fold lower, respectively, than that on 30S IC_wT_* (Supplementary Table 2). It is notable that these estimated decreases in the occupancies of wtIF2(GTP) on 30S IC_wT,Met_* and 30S IC_wT,OH_* closely approximate the decreases in the rates of 50S subunit joining to 30S IC_wT,Met_* and 30S IC_wT,OH_*. Thus, it is possible that the lack of an *N*-formyl moiety or *N*-formyl-methionine on 30S IC-bound Met-tRNA^fMet^ or tRNA^fMet^ decreases the rate of 50S subunit joining by reducing the occupancy of wtIF2(GTP) on these pseudo 30S ICs to ~19% and ~9%, respectively (Supplementary Table 2), rather than by stabilizing wtIF2(GTP) in an inactive conformation at an occupancy of nearly 100% on these pseudo 30S ICs, as we have previously suggested^21^. The key question therefore becomes whether the activation of IF2(GTP) for rapid 50S subunit joining merely involves an fMet-tRNA^fMet^-dependent increase in the affinity of IF2(GTP) for the 30S IC or whether, in addition, there is an fMet-tRNA^fMet^-dependent change in the conformation of 30S IC-bound IF2(GTP).

**Fig. 4:**
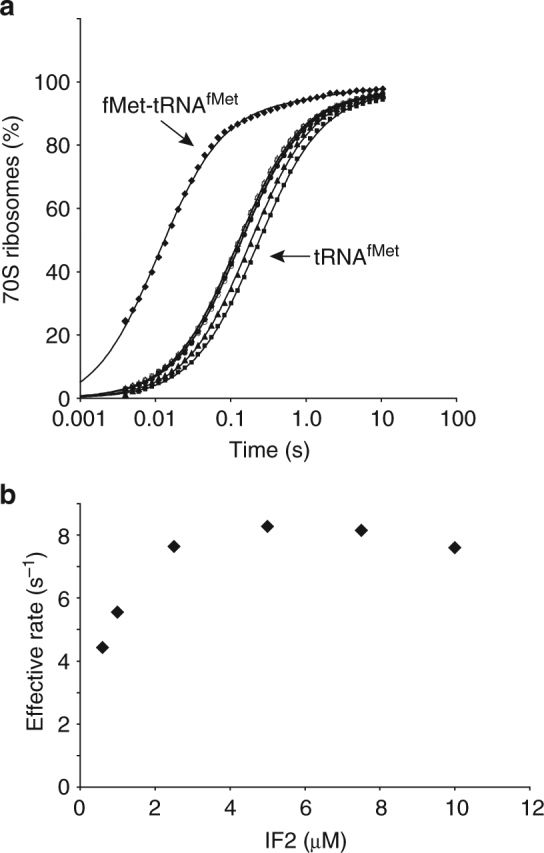
Effect of IF2 concentration on the rate of 50S subunit joining to a pseudo 30S IC. **a** Ensemble kinetics of 70S IC formation after rapid mixing of 50S subunits with 30S IC_wT_ assembled in the presence of 1 μM wtIF2 or 30S IC_wT,OH_s assembled in the presence of 0.6–10 μM wtIF2. **b** Effective rates of 50S subunit joining to 30S IC_wT,OH_s containing increasing concentrations of wtIF2

To address this question, we performed ensemble kinetic experiments to measure the rate of 50S subunit joining to 30S IC_wT,OH_ as a function of wtIF2(GTP) concentrations that were high enough to saturate 30S IC_wT,OH_ with wtIF2(GTP). As a reference, we measured the maximal rate of 50S subunit joining to 30S IC_wT_ using a wtIF2(GTP) concentration of 1.0 µM and obtained a rate of ~80 s^−1^ (Fig. 4), a result that, in excellent agreement with our previous studies^21^, is ~14-fold faster than the rate of 50S subunit joining to 30S IC_wT,OH_ measured at the same wtIF2(GTP) concentration. Titrating the concentration of wtIF2(GTP) from 0.6 to 10 µM using 30S IC_wT,OH_ resulted in a small, ~1.5-fold increase in the rate of 50S subunit joining, suggesting that, at the 0.6 µM concentrations of wtIF2(GTP) used in the previous studies, 30S IC_wT,OH_ was not saturated with wtIF2(GTP). Nonetheless, we find that the rate of 50S subunit joining to 30S IC_wT,OH_ plateaus at a wtIF2(GTP) concentration of ~2.5 µM, indicating that at wtIF2(GTP) concentrations above ~2.5 µM, 30S IC_wT,OH_ is saturated with wtIF2(GTP). Interestingly, we find that, even when 30S IC_wT,OH_ is saturated with wtIF2(GTP), the rate of 50S subunit joining is still ~11-fold lower than the maximal rate of 50S subunit joining to 30S IC_wT_. Based on these results, we conclude that the decreased rate of 50S subunit joining originates from a conformation of 30S IC_wT,OH_-bound wtIF2(GTP) that is inactive for rapid 50S subunit joining.

### The conformation of dI-dIII confers rapid subunit joining

To investigate whether and how the activating mutation in dIII modulates the affinity of IF2(GTP) for pseudo 30S ICs and the conformation of the resulting pseudo 30S IC-bound IF2(GTP), we performed smFRET experiments using mutIF2[Cy5]_dIV_(GTP) and 30S IC_mT,Met_ or 30S IC_mT,OH_. The results of these experiments demonstrate that excursions to the mutIF2(GTP)-bound state in the 30S IC_mT,Met_ and 30S IC_mT,OH_ *E*_FRET_ trajectories are significantly longer than those to the wtIF2(GTP)-bound states in the 30S IC_wT,Met_ and 30S IC_wT,OH_ *E*_FRET_ trajectories (compare Fig. 3c, d with Fig. 3a, b). Consistent with this, we find that *K*_d,mT,Met_ and *K*_d,mT,OH_ are ~20-fold and ~30-fold smaller than *K*_d,wT,Met_ and *K*_d,wT,OH_, respectively (Table 1). The activating mutation in dIII therefore enables mutIF2(GTP) to bind to 30S IC_mT,Met_ and 30S IC_mT,OH_ with an affinity that is over an order of magnitude higher than that with which wtIF2(GTP) binds to 30S IC_wT,Met_ and 30S IC_wT,OH_. This demonstrates that high-affinity binding of IF2(GTP) to the 30S IC does not necessarily require dIV to establish direct interactions with the *N*-formyl moiety or *N*-formyl-methionine of the P site-bound fMet-tRNA^fMet^. Rather, it is the conformation of dI-dIII that contributes significantly to the affinity of IF2(GTP) for the 30S IC. Such a contribution could arise from direct interactions between dIII and S12 or some other component of the 30S IC and/or from allosteric modulation of the interactions that dII makes with h5 and h14 of 16S rRNA.

Interestingly, the distribution of *E*_FRET_ values for the mutIF2(GTP)-bound state of 30S IC_mT,Met_ and 30S IC_mT,OH_ are very similar to those for the wtIF2(GTP)-bound state of 30S IC_wT,Met_ and 30S IC_wT,OH_. Specifically, the distribution of *E*_FRET_ values for the mutIF2(GTP)-bound state of 30S IC_mT,Met_ (Fig. 3c and Supplementary Fig. 5f) exhibited two non-zero *E*_FRET_ peaks. The first peak encompassed a smaller, 42 ± 5.7%, subpopulation of the mutIF2(GTP)-bound state of 30S IC_mT,Met_ and was centered at an <*E*_FRET_> of 0.83 ± 0.04 that is within error of that observed for the larger subpopulation of the wtIF2(GTP)-bound state of 30S IC_wT,Met_ (*p* value = 0.7, Supplementary Table 1). The second peak encompassed a larger, 58 ± 5.7%, subpopulation of the mutIF2(GTP)-bound state of 30S IC_mT,Met_ and was centered at an <*E*_FRET_> of 0.57 ± 0.02 that is also within error of that observed for the smaller subpopulation of the wtIF2(GTP)-bound state of 30S IC_wT,Met_ (*p* value = 0.4, Supplementary Table 1). Similarly, the distribution of *E*_FRET_ values for the mutIF2(GTP)-bound state of 30S IC_mT,OH_ (Fig. 3d and Supplementary Fig. 5h) exhibited a single non-zero *E*_FRET_ peak centered at an <*E*_FRET_> of 0.57 ± 0.01 that is within error of that observed for the larger subpopulation of the mutIF2(GTP)-bound state 30S IC_mT,Met_ and the wtIF2(GTP)-bound state of 30S IC_wT,OH_ (*p* value = 0.8, Supplementary Table 1). The fact that the <*E*_FRET_> s that we observe for 30S IC_mT,Met_- and 30S IC_mT,OH_-bound mutIF2(GTP), here are within error of the <*E*_FRET_> s observed for 30S IC_wT,Met_- and 30S IC_wT,OH_-bound wtIF2(GTP) strongly suggests that the activating mutation in dIII does not significantly alter the positions of dIV of mutIF2(GTP) in 30S IC_mT,Met_ and 30S IC_mT,OH_ relative to those of dIV of wtIF2(GTP) in 30S IC_wT,Met_ and 30S IC_wT,OH_. Indeed, with the exception of a relatively small, ~15%, shift in the subpopulation occupancies of the IF2(GTP)-bound states of 30S IC_wT,Met_ and 30S IC_mT,Met_, dIV of wtIF2(GTP) and mutIF2(GTP) seem to adopt similar conformations in 30S ICs carrying Met-tRNA^fMet^ and tRNA^fMet^.

Previously, we have used ensemble kinetic experiments to show that the rates of 50S subunit joining to 30S IC_mT,Met_* and 30S IC_mT,OH_* are approximately fourfold and ~12-fold higher than to 30S IC_wT,Met_* and 30S IC_wT,OH_*, respectively^21^. Thus, the activating mutation in dIII enables mutIF2(GTP) to catalyze 50S subunit joining to 30S IC_mT,Met_* and 30S IC_mT,OH_* at rates that are within 30% of those observed for 30S IC_wT_* and 30S IC_mT_*. Based on the results reported here, we propose that the activating mutation in dIII enables this increase in the rate of subunit joining by stabilizing a conformation of dI-dIII, that not only increases the affinity of mutIF2(GTP) for 30S IC_mT,Met_ and 30S IC_mT,OH_, but that is optimized for the rapid recruitment^35,36^ and/or docking of the 50S subunit onto 30S IC_mT,Met_ and 30S IC_mT,OH_. We speculate that, in the context of wtIF2(GTP), this conformation of dI-dIII is rendered conditional on the direct interactions of dIV with the *N*-formyl-methionine.

## Discussion

**Fig. 5:**
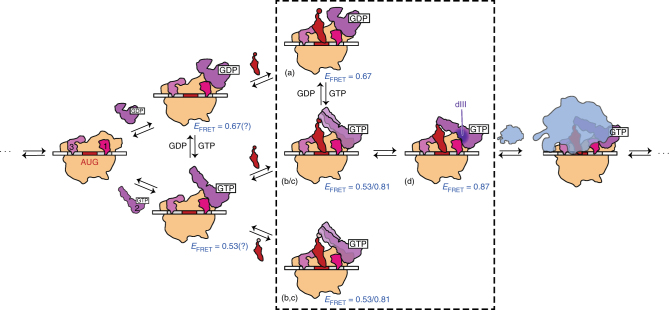
Structural model for the GTP and fMet-tRNA^fMet^-dependent activation of 30S IC-bound IF2. 30S IC-bound IF2 can occupy at least four distinct conformational states relative to the P-site tRNA (denoted as conformations *a*–*d*). These conformational states are characterized by *E*_FRET_ values of 0.67 (*a*), 0.53 (*b*), 0.81 (*c*), and 0.87 (*d*). The dotted box highlights 30S ICs and pseudo 30S ICs studied in this work and their corresponding *E*_FRET_ values. 30S ICs and *E*_FRET_ values indicated outside of the dotted box are predicted conformational states of IF2. (Central panel) The specific binding of GTP to dI of IF2 is allosterically communicated through dIII and results in a repositioning of dIV closer to the P site of the 30S IC and further from dI-III. The specific recognition of the *N*-formyl-methionine of a P-site-bound fMet-tRNA^fMet^ by dIV of IF2(GTP) feeds back to dIII (dark purple), thereby stabilizing a conformation of dI-dIII of IF2 that is active for rapid 50S subunit joining. In contrast, (top panel) the binding of GDP to dI of IF2, or (bottom panel) the presence of an unformylated Met-tRNA^fMet^, or an elongator tRNA in the P site fails to stabilize the active conformation of IF2, instead leaving IF2 in a conformation(s) that are inactive for rapid 50S subunit joining

Here, we have used a combination of smFRET and ensemble kinetic studies of 50S subunit joining to elucidate the mechanism of IF2 activation for rapid 50S subunit joining to the 30S IC. Our results demonstrate how GTP and fMet-tRNA^fMet^ stabilize the specific conformation of 30S IC-bound IF2 that confers high-affinity binding to the 30S IC and rapid 50S subunit joining. Based on our findings, we propose a model for IF2 activation during translation initiation (Fig. 5). In this model, four conformations of 30S IC-bound IF2, which we denote as conformations *a*–*d* in Fig. 5, play major roles. In conformation *a*, dI is not in the GTP-bound form, dII and dIII do not interact fully with the 30S subunit, and dIV is positioned closer to dI-dIII than to the P site-bound tRNA. This conformation corresponds to the <*E*_FRET_> of 0.67 ± 0.01 that we observe for 30S IC_wD_-bound wtIF2(GDP) and it is inactive for rapid 50S subunit joining. In conformation *b*, dI is in the GTP-bound form, dII and dIII have established increased interactions with the 30S subunit, and dIV is positioned closer to the P site-bound tRNA than to dI-dIII. This GTP-dependent repositioning of dIV is driven by an allosteric mechanism in which binding of GTP to dI triggers a conformational change and/or repositioning of dI-dIII, which ultimately places dIV closer to the P site. Independent evidence in favor of our model comes from structural studies in which binding of GTP to dI results in a restructuring of dI-dIII that has been predicted to modulate the interactions of dII with h14 and h5 of 16S rRNA^37^ and of dIII with S12^27^. In conformation *b*, which corresponds to the <*E*_FRET_> value of 0.53 ± 0.02 that we observe for 30S IC_wT,OH_-bound wtIF2(GTP), dIV does not make any stabilizing contacts with fMet-tRNA^fMet^. Conformation *c*, which corresponds to the <*E*_FRET_> of 0.81 ± 0.01 that we observe in 30S IC_wT,Met_-bound wtIF2(GTP), is similar to the second conformation, except that dIV now makes partial interactions with the *N*-formyl-methionine of a P-site fMet-tRNA^fMet^. In conformation *d*, which corresponds to the <*E*_FRET_> of 0.87 ± 0.02 that we observe in 30S IC_wT_-bound wtIF2(GTP), dIV interacts fully with the *N*-formyl-methionine of the P-site fMet-tRNA^fMet^ and has adopted a position that allosterically feeds back and stabilizes the conformation of dI-dIII that is active for rapid 50S subunit joining. Independent evidence in support of the idea that the interactions between dIV and the *N*-formyl-methionine allosterically feed back to dI-dIII in order to stabilize a conformation that is active for rapid 50S subunit joining comes from previous measurements of the affinity of dI for GTP in which it was observed that the presence of a P-site fMet-tRNA^fMet^ allosterically increases the affinity of dI for GTP^38^.

A major finding of this study is that dIII integrates GTP binding to dI and fMet-tRNA^fMet^ recognition by dIV in order to allosterically regulate the affinity of IF2 for the 30S IC and the conformation of the resulting 30S IC-bound IF2. This suggests that dIII may serve as a target for antibiotics that inhibit IF2 allosterically. To explore this option, further characterization of the mechanism of IF2 activation and, in particular, the dynamic interplay between dI-dIV will be necessary. This will require the development of additional, intramolecular labeling schemes to directly probe the interdomain dynamics of IF2. In addition, while low- to high-resolution cryo-EM structures of the active conformation of 30S IC-bound IF2 have been reported^16–18^, a comprehensive structural understanding of IF2 activation will undoubtedly require not only higher resolution cryo-EM or crystallographic structures of the active conformation of 30S IC-bound IF2, but also the inactive conformation(s) of 30S IC-bound IF2 (e.g., 30S IC_wD_, 30S IC_wT,Met_, or 30S IC_wT,OH_).

## Methods

### Preparation and fluorescence labeling of IFs

pProEX-HTb expression vectors containing a cloned copy of the *E. coli* IF1 gene or a cloned copy of the *E. coli* IF2 gene (γ-isoform) downstream of a six-histidine (6xHis) tag were transformed into the BL21(DE3) strain and purified as described previously^28,39^. Briefly, transformed bacteria was grown in Terrific Broth at 37 °C until the bacterial cell culture reached an optical density (OD_600_) of ~0.8. Overexpression of the IFs was induced by the addition of 1 mM Isopropyl β-d-1-thiogalactopyranoside and the induced cultures were grown for an additional 2–4 h at 37 °C. 6xHis-tagged IF1 and IF2 were purified by Ni^2+^-nitrilotriacetic acid affinity chromatography using the batch procedure (Qiagen). Subsequently, the N-terminal 6xHis tags were removed by incubating the purified IFs with the tobacco etch virus protease. Further purification of untagged IF1 was achieved using a HiLoad 16/60 Superdex 75 prep grade gel filtration column and further purification of untagged IF2 was achieved using a HiTrap SP HP cation exchange column. In both instances, peak fractions were collected, concentrated using an Amicon centrifugal filtration device, and the IFs were buffer exchanged into 2× IF storage buffer (20 mM Tris-OAc(pH_RT_ = 6.9), 100 mM KCl, 20 mM Mg(OAc)_2_, and 10 mM βME). Following the addition of 1 volume of 100% glycerol, the purified IFs were stored at –20 °C. The concentration of IF1 was measured using the Bradford assay, and the concentration of IF2 was determined by measuring its ultraviolet absorbance at 280 nm and by using its molar extinction coefficient (27390 M^−1^ cm^−1^), which was calculated using the ProtParam tool on the ExPASy Proteomics Server.

Mutant variants of IF2 harboring either (1) a serine-to-tyrosine mutation at amino acid 753 (IF2(Ser753Tyr)), (2) a glycine-to-cysteine mutation at amino acid position 810 (IF2(Gly810Cys)), (3) a Gly810Cys and a Ser753Tyr mutation (IF2(S753Y-G810C)), or (4) an alanine-to-cysteine mutation at amino acid position 760 (IF2(Ala760Cys)) were generated using the mutagenic DNA primers shown below and the PfuUltra High-Fidelity DNA Polymerase (Agilent Technologies) following the manufacturer’s protocol.

Gly810Cys:

5′-CGCCGAAATTTTGTGCCATCGCAGGCTGTATG-3′

5′-CATACAGCCTGCGATGGCACAAAATTTCGGCG-3′.

Ser753Tyr:

5′-GCTTTAACGTACGTGCTGATGCCTAT-3′

5′-CCGCTTCAATCACTTTACGTGCATAG-3′.

Ala760Cys:

5′-CTCTGCACGTAAAGTGATTGAATGCGAA-3′

5′-CGCAGATCCAGGCTTTCGCATTCAATCAC-3′.

The mutant IF2 constructs were verified by automated DNA sequencing. The overexpression and purification of these IF2 variants was performed exactly as described above for wild-type IF2.

The cysteine residue introduced into amino acid position 810 (cysteine-810) in the IF2(Gly810Cys) and IF2(Ser753Tyr-Gly810Cys) variants or into amino acid position 760 (cysteine-760) in the IF2(Ala760Cys) variant was used to label IF2 with the maleimide-derivatized Cyanine 5, or Cy5, fluorophore as described previously^11,28^ and generate wtIF2[Cy5]_dIV_ and mutIF2[Cy5]_dIV_ or wtIF2[Cy5]_dIII_, respectively. Briefly, IF2(Gly810Cys), IF2(Ser753Tyr-Gly810Cys), and IF2(Ala760Cys) were buffer exchanged into labeling buffer containing a 10-fold molar excess of tris(2-carboxyethyl)phosphine hydrochloride using a Micro Bio-Spin P6 gel filtration spin column and incubated at room temperature for 30 min. To specifically and quantitatively label IF2 at either cysteine-810 and cysteine-760, a 10-fold molar excess of Cy5 dissolved in anhydrous dimethylsulfoxide was added to the labeling reaction and incubated overnight at 4 °C. Cy5-labeled IF2 was separated from free, unreacted Cy5 using gel filtration chromatography. The labeling efficiency of IF2 at cysteine-810 (~90%) and IF2 at cysteine-760 (~80%) was determined from the ratio of the concentration of IF2 and the concentration of Cy5. The concentration of Cy5 was determined by measuring its absorbance at 650 nm and using its molar extinction coefficient (250,000 M^−1^ cm^−1^). The concentration of IF2 was corrected for the 5% absorbance of Cy5 at 280 nm.

### Preparation of 30S subunits

Highly active 30S subunits were purified from the *E. coli* strain MRE600 as described previously^28^. Briefly, clarified cell lysates were centrifuged through a sucrose cushion solution (20 mM Tris-HCl (pH_4 °C = _7.2), 500 mM NH4Cl, 10 mM MgCl_2_, 0.5 mM EDTA, 6 mM βME, 37.7% sucrose) to isolate crude ribosomes. Next, crude ribosomes were centrifuged through a 10–40% sucrose density gradient to isolate tight-coupled 70S ribosomes. To promote the dissociation of tight-coupled 70S ribosomes into 30S and 50S ribosomal subunits, the tight-coupled 70S ribosomes were dialyzed into ribosome dissociation buffer (10 mM Tris-OAc (pH_4 °C_ = 7.5), 60 mM NH_4_Cl, 1 mM MgCl_2_, 0.5 mM EDTA, 6 mM βME). To isolate 30S subunits, the dissociated ribosomes were centrifuged through a 10–40% sucrose gradient prepared in ribosome dissociation buffer. Purified 30S subunits were pelleted by ultra-centrifugation, re-suspended in ribosome storage buffer (10 mM Tris-OAc (pH_4 °C_ = 7.5), 60 mM NH_4_Cl, 7.5 mM MgCl_2_, 0.5 mM EDTA, 6 mM βME), quantified by measuring the ultraviolet absorbance at 260 nm (1 A_260_ unit = 79 nM), and stored in small aliquots at –80 °C.

### Preparation of mRNAs

The 5′-biotinylated mRNA used in our single molecule experiments (5′-bio-mRNA_AUG_) and the mRNA used in our primer-extension inhibition, or “toeprinting”-based IF2 activity assay (mRNApri-ext) were both variants of the mRNA encoding gene product 32 from T4 bacteriophage. 5′-bio-mRNA_AUG_ was chemically synthesized and mRNApri-ext was generated by in vitro transcription using T7 RNA polymerase as previously described^28^. The mRNA sequence for 5′-bio-mRNA_AUG_ and mRNApri-ext are shown below. In both of the mRNA sequences, the Shine–Dalgarno (SD) sequence is underlined, the start codon is underlined and bolded, and the spacer sequence between the SD sequence and the start codon is italicized. In addition, for mRNApri-ext, the primer annealing site used to reverse transcribe the message is underlined, bolded, and italicized. The mMFTI mRNA used in our light-scattering experiments is also shown below and the SD sequence, start codon, and spacer sequence are highlighted as indicated for 5′-bio-mRNA_AUG_ and mRNApri-ext.

5′-bio-mRNA_AUG_:

5′-biotin-CAACCUAAAACUUACACAAAUUAAAAAGGAAAU *AGAC* AUGUUCAAAGUCGAAAAAUCUACUGCU-3′.

mRNApri-ext:

5′-GGCAACCUAAAACUUACACAGGGCCCUAAGGAAAU *AAAA* AUGUUUAAAGAAGUAUACAC UGCUGAACUCGCUGCACAAAUGGCUAAACUGAAUGGCAAUAAAGGUUUUUCUUCUGAAGAUAAAGGCGAGUGGAAACUGAAACUCGAUAAUGCGGGUAACGGUCAAGCAGUAAUUCGUUUUCUUCCGUCUAAAAAUGAUGAACAAGCACCAUUCGCAAUUCUUGUAAAUCACGGUUUCAAGAAAAAUGGUAAAUGGUAUAUUGAAACAUGUUCAUCUACCCAUGGUGAUUACGAUUCUUGCCCAGUAUGUCAAUACAUCAGUAAAAAUGAUCUAUACAACACUGACAAUAAAGAGUACAGUCUUGUUAAACGUAAAACUUCUUACUGGGCUAACAUUCUUGUAGUAAAAGACCCAGCUGCUCCAGAAAACGAAGGUAAAGUAUUUAAAUACCGUUUCGGUAAGAAAAUCUGGGAUAAAAUCAAUGCAAUGAUUGCGGUUGAUGUUGAAAUGGGUGAAACUCCAGUUGAUGUAACUUGUCCGUGGGAAGGUGCUAACUUUGUACUGAAAGUUAAACAAGUUUCUGGAUUUAGUAACUACGAUGAAUCUAAAUUCCUGAAUCAAUCUGCGAUUCCAAACAUUGACGAUGAAUCUUUCCAGAAAGAACUGUUCGAACAAAUGGUCGACCUUUCUGAAAUGACUUCUAAAGAUAAAUAAGG-3′.

mMFTI mRNA:

5′-GGGAAUUCGGGCCCUUGUUAACAAUUAAGGAGGU *AUACU* AUGUUUACGAUUUAAUUGCAGAAAAAAAAAAAAAAAAAAAAA-3′.

### Preparation and fluorescence labeling of tRNAs

tRNA^fMet^ was aminoacylated and formylated using purified methionyl-tRNA synthetase and methionyl-tRNA formyltransferase following previously published protocols^39^. The aminoacylation and formylation efficiency was >90% as determined after separating the tRNA^fMet^, Met-tRNA^fMet^, and fMet-tRNA^fMet^ species using a TSKgel Phenyl-5PW hydrophobic interaction chromatography (HIC) column. tRNA^fMet^ was labeled by reacting its naturally occurring 4-thiouridine at nucleotide position 8 with a maleimide-derivatized cyanine 3, or Cy3, as described previously^28^. Cy3-labeled tRNA^fMet^ was separated from free, unreacted Cy3 by extensive phenol extraction, and from unlabeled tRNA^fMet^ using HIC chromatography.

### Preparation of 30S ICs

For smFRET experiments, 30S ICs were assembled by combining 0.6 μM Cy3-labeled initiator tRNA (fMet-tRNA(Cy3)^fMet^, Met-tRNA(Cy3)^fMet^, or tRNA(Cy3)^fMet^), 0.9 μM IF1, 0.9 μM Cy5-labeled wildtype or mutant IF2 (wtIF2[Cy5]_dIV_, mutIF2[Cy5]_dIV_, or wtIF2[Cy5]_dIII_), 1.8 μM 5′-biotinylated mRNA and 0.6 μM purified *E*. *coli* 30S subunits in Tris-polymix buffer (50 mM Tris-OAc (pH_RT_ of 7.5), 100 mM KCL, 5 mM NH_4_OAc, 5 mM Mg(OAc)_2_, 0.1 mM EDTA, 1 mM GTP (or 1 mM GDP), 5 mM putrescine-HCl, 1 mM spermidine-free base, and 6 mM βME. Reactions were incubated at 37 °C for 10 min and transferred to ice for an additional 5 min. Small aliquots were prepared, flash-frozen with liquid nitrogen, and stored at −80 °C.

For the toeprinting experiments, 30S ICs were assembled in a Tris-Polymix buffer containing 3 mM Mg(OAc)_2_ as described previously^28,39^. Briefly, 0.5 μM 30S subunits, 5 μM of the indicated IF2 variant, and 0.8 mM GTP were combined and incubated for 10 min at 37 °C. Next, 0.25 μM of mRNApri-ext pre-annealed with a 5′[^32^P]-labeled DNA primer of sequence TATTGCCATTCAGTTTAG was added to the reaction and incubated for an additional 10 min at 37 °C. The DNA primer was radioactively labeled with [γ-^32^P]ATP and T4 polynucleotide kinase (New England Biolabs) using the manufacturer’s protocol. Finally, 0.8 μM fMet-tRNA^fMet^, and/or tRNA^Phe^ was added to the reaction and incubated for an additional 10 min at 37 °C.

For light-scattering experiments, 30S ICs were assembled in 4′(2-hydroxyethyl)-1-piperazineethanesulfonic acid (HEPES)-Polymix buffer (30 mM HEPES pH_RT=7.5_, 95 mM KCl, 5 mM NH_4_Cl, 0.5 mM Calcium chloride (CaCl_2_), 8 mM putrescine-HCl, 1 mM spermidine free base, 6 mM Mg(OAc)_2_, 2 mM phosphoenolpyruvate, 1 mM GTP, 1 mM ATP, 1 μg ml^−1^, pyruvate kinase, and 1 μg ml^−1^ of myokinase) as described previously^21^. Briefly, 0.32 μM 30S subunits were combined with 0.8 μM mMFTI mRNA, 1 μM IF1, either 0.6 μM IF2 (Supplementary Fig. 3), or 0.6–10 μM IF2 (Fig. 4), and either 0.9 μM fMet-tRNA^fMet^, or 1.6 μM deacylated tRNA^fMet^. Reactions were incubated for 20 min at 37 °C. Note that all concentrations indicated represent final concentrations after rapid mixing with 50S subunits.

### smFRET experiments

30S ICs were tethered to the polyethylene glycol (PEG)/biotinylated-PEG-derivatized surface of a quartz microfluidic flowcell via a biotin-streptavidin-biotin bridge and imaged it at single-molecule resolution using a lab-built, wide-field, prism-based TIRF microscope. The Cy3 fluorophore was directly excited using a 532-nm, diode-pumped, solid-state laser (CrystaLaser) under a ~12 mW excitation laser power, as measured at the prism. The Cy3 and Cy5 fluorescence emissions were simultaneously collected by a 1.2 numerical aperture/×60 water-immersion objective (Nikon) and wavelength separated using a two channel, simultaneous-imaging system (DV2, Photometrics Inc.). The Cy3 and Cy5 fluorescence emissions were imaged at an acquisition time of 0.1 s per frame using a 512 × 512 pixel, back-illuminated EMCCD camera (Cascade II:512, Photometrics Inc.) operating with 2 pixel × 2 pixel binning. As before^28^, we supplemented all buffers with 1 mM GTP, ~1 µM IF1, and 25 nM wtIF2[Cy5]_dIV_, mutIF2[Cy5]_dIV_, or wtIF2[Cy5]_dIII_ in order to allow these IFs to re-associate with 30S ICs from which they might have dissociated during tethering and/or TIRF imaging.

The Cy3 and Cy5 fields of view from each individual movie were aligned using custom-built software written in Java, and co-localized Cy3 and Cy5 fluorescence signals were used to plot raw, Cy3 and Cy5, fluorescence intensity vs. time trajectories. The Cy5 fluorescence intensity in these trajectories was corrected for bleed-through of Cy3 emission into the Cy5 channel (~7%) and the trajectories were baseline-corrected using custom scripts written in MATLAB. The raw, bleed-through- and baseline-corrected Cy3 and Cy5 fluorescence intensity vs. time trajectories that exhibited anti-correlation of the Cy3 and Cy5 fluorescence signals and either (i) single-step photobleaching of the Cy3 and/or Cy5 fluorophores, or (ii) average Cy3 and Cy5 fluorescence intensities characteristic of single Cy3 and Cy5 fluorophores, were kept for further analysis. *E*_FRET_ vs. time trajectories (hereafter *E*_FRET_ trajectories) were obtained from the raw, bleed-through- and baseline-corrected Cy3 and Cy5 trajectories by calculating the *E*_FRET_ value at each time point of the trajectory. The *E*_FRET_ values were calculated by dividing the Cy5 fluorescence intensity (*I*_Cy5_) by the sum of the Cy3 and Cy5 fluorescence intensities (*I*_Cy5 +_ *I*_Cy3_). The *E*_FRET_ trajectories were truncated after photobleaching of Cy3.

Kinetic parameters describing the interaction of IF2 with the 30S IC was extracted from the *E*_FRET_ trajectories using a previously described^28^ transition probability matrix-based population decay analysis. The *E*_FRET_ trajectories from three independent data sets were idealized to a HMM using the vbFRET software package^40^. Subsequently, the idealized *E*_FRET_ trajectories were used to construct a 2 × 2 counting matrix with matrix elements *n*_*ij*_, where *i* represents the IF2-free state of the 30S IC (*E*_FRET_ ≤ 0.2) and *j* represents the IF2-bound state of the 30S IC (*E*_FRET_ > 0.2). The row elements of the counting matrix were normalized to construct a 2 × 2 transition probability matrix with matrix elements p_*ij*_. The diagonal elements of the transition probability matrix (p_*ii*_ and p_*jj*_) report the probability of staying in the IF2-free state (p_*ii*_) of the 30S IC and the probability of staying in the IF2-bound state (p_*jj*_) of the 30S IC, given a discreet time interval (*τ*), which is set by the time resolution of our smFRET experiments (0.1 s per frame). The state transition probabilities, p_*ii*_ and p_*jj*_, were converted to the rate of IF2 association (*k*_*a*_) to the 30S IC and the rate of IF2 dissociation (*k*_*d*_) from the 30S IC, respectively, using the following two equations:

$${k_a} = -{\rm{ln}}\left( {{p_{{\rm{IF2 - free}} \to {\rm{IF2 - free}}}}} \right)/\left[ {{\rm{IF}}2} \right]t\,{\rm{and}}\,{k_d} = -{\rm{ln}}\left( {{p_{{\rm{IF2 - bound}} \to {\rm{IF2 - bound}}}}} \right)/t$$

The transition probability matrix-based population decay analysis described in the previous paragraph was used to correct for the effects of Cy5 photobleaching in the *E*_FRET_ trajectories from 30S IC_wT_ and 30S IC_mT_ and to correct for the effects of Cy3 photobleaching in the *E*_FRET_ trajectories from 30S IC_wT,Met_ and 30S IC_wT,OH_. The exceedingly stable and long-lived binding of wtIF2 and mutIF2 to 30S IC_wT_ and 30S IC_mT_ (Fig. 1a, b) results in a significant number of *E*_FRET_ trajectories in which IF2 remains stably bound to the 30S IC after Cy5 photobleaching. The failure to account for this in these *E*_FRET_ trajectories results in an aberrantly high probability of remaining in the IF2-free state and thus a significant overestimation of *k*_d_. Therefore, to correct for the effects of Cy5 photobleaching on *k*_d_, the final dwell of each of the *E*_FRET_ trajectories that exhibited an *E*_FRET_ ≤ 0.2 was not included in our analysis.

On the other hand, the exceedingly rare and short-lived transitions to the IF2-bound state that are observed for the interaction of wtIF2(GTP) with pseudo 30S ICs (30S IC_wT,Met_ and 30S IC_wT,OH_, Fig. 3a and b, respectively), result in a subpopulation of *E*_FRET_ trajectories originating from pseudo 30S ICs that are capable of undergoing an IF2 binding event, but fail to do so during our observation window. The failure to account for this subpopulation of *E*_FRET_ trajectories, which represent long dwells comprised of transitions in the IF2-free state of the 30S IC, results in a decrease in the probability of remaining in the IF2-free state and thus an overestimation of *k*_a_. To calculate corrected values of *k*_a_ for 30S IC_wT,Met_ and 30S IC_wT,OH_, we generated a series of simulated *E*_FRET_ trajectories with an *E*_FRET_ value of zero and pooled these with the observed *E*_FRET_ trajectories. The number of simulated *E*_FRET_ trajectories for each pseudo 30S IC was determined by taking the number of observed *E*_FRET_ trajectories that exhibited at least one IF2 binding event in 30S IC_wT,Met_ or 30S IC_wT,OH_ and multiplying by a correction factor given by:

$${\rm{Correction}}\,{\rm{factor}}\,{\rm{(Cf)}} = \frac{{{\rm{\% }}\,{\rm{capable}} - {\rm{\% }}\,{\rm{observed}}}}{{{\rm{\% }}\,{\rm{observed}}}},$$

where “% capable” represents the fraction of *E*_FRET_ trajectories that exhibited at least one binding event in the analogous 30S IC formed with fMet-tRNA^fMet^ (30S IC_wT_), and “% observed” represents the fraction of *E*_FRET_ trajectories that exhibited at least one binding event in 30S IC_wT,Met_ or 30S IC_wT,OH_. The length of the simulated *E*_FRET_ trajectories was set to the average length of the observed *E*_FRET_ trajectories for either 30S IC_wT,Met_ or 30S IC_wT,OH_ prior to Cy3 photobleaching.

### Ensemble toeprinting and kinetic experiments

The primer-extension inhibition-, or “toeprinting”-based IF2 activity assay reports on the position of the 30S IC on mRNApri-ext with single-nucleotide resolution and thus measures the ability of IF2 to direct the selection of the authentic initiator fMet-tRNA^fMet^ into the P site of the 30S IC, over elongator tRNAs. Primer extension was performed using the Avian Myeloblastosis (AMV) reverse transcriptase as described previously^28,39^. Briefly, 25 μl reactions containing 5 μl of the various 30S ICs, 1.2 mM ATP, 0.5 mM of each dNTP, and 6 units of AMV and Tris-Polymix with 10 mM Mg(OAc)_2_ were incubated at 37 °C for 15 min. Following phenol–chloroform extraction and ethanol precipitation, complementary DNA (cDNA) pellets were re-suspended in formamide loading buffer, heat denatured at 95 °C for 5 min, and cDNA fragments were resolved on a 9% sequencing gel.

Rayleigh light scattering-based ensemble kinetic 50S subunit joining experiments were performed as described previously^21^. Briefly, 0.6–0.8 ml of 0.36 μM 50S subunits and 0.6–0.8 ml mixture containing 0.36 μM 30S IC assembled with IF1, fMet-tRNA^fMet^, or tRNA^fMet^ (or no tRNA) and IF2 (wildtype or mutant, added in different concentrations) were pre-incubated for 20 min at 37 °C and loaded into the syringes of our stopped-flow instrument (SX-20 Applied Photophysics, Leatherhead, UK). The kinetics of 70S IC formation was monitored at 37 °C with light scattering after rapid mixing of equal volumes of the 30S IC mixture and the 50S subunits.

### Data availability

The data that support the findings of this study are available from the corresponding author upon request.

## Electronic supplementary material

Supplementary Information
