# Supplementary material for: A conformational switch in initiation factor 2 controls the fidelity of translation initiation in bacteria
Source: Nat Commun. 2017 Nov 14;8:1475. doi: 10.1038/s41467-017-01492-6 (PMC5684235; doi:10.1038/s41467-017-01492-6)
Supplement: Supplementary file 1 — Supplementary Information [file 41467_2017_1492_MOESM1_ESM.pdf]

SUPPLEMENTARY INFORMATION

A conformational switch in initiation factor 2 controls the fidelity of translation initiation in bacteria

Kelvin Caban[1], Michael Pavlov[2], Måns Ehrenberg[2], and Ruben L. Gonzalez, Jr.[1,*]

[1]*Department of Chemistry, Columbia University, 3000 Broadway, MC3126, New York, NY 10027, USA*

[2]*Department of Cell and Molecular Biology, BMC, Uppsala University, Husargatan 3, Uppsala, Sweden 751 24*

*To whom correspondence should be addressed: Ruben L. Gonzalez Jr., Department of Chemistry, Columbia University, 3000 Broadway, MC3126, New York, NY 10027, USA, Tel.: (212) 854-1096; Fax: (212) 932-1289; Email: rlg2118@columbia.edu

Supplementary Figures

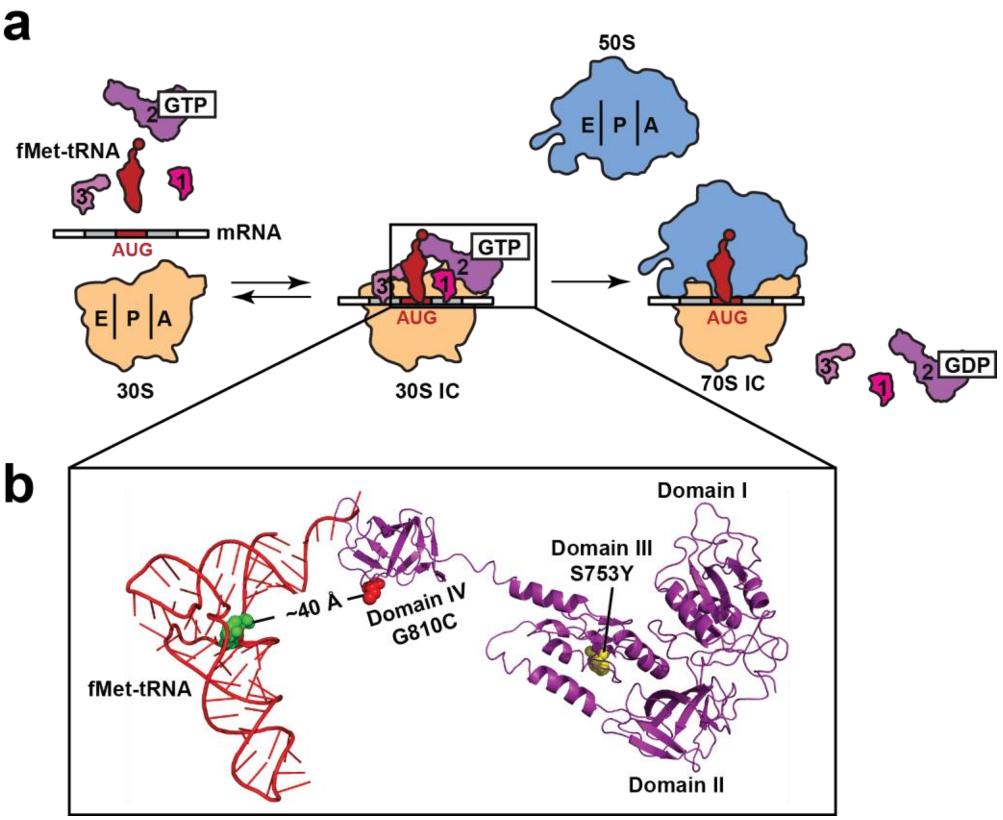

Supplementary Figure 1: The bacterial translation initiation pathway. (a) Cartoon representation of the translation initiation pathway. Translation initiation begins with the assembly of a 30S initiation complex (30S IC) consisting of the 30S ribosomal subunit, mRNA, IFs 1-3, and initiator fMet-tRNA^{fMet}. Joining of the 50S ribosomal subunit to the 30S IC and the subsequent departure of the IFs gives rise to the elongation-competent 70S IC. (b) Structural model of the IF2•tRNA sub-complex depicting the IF2-tRNA smFRET signal (pdb 1Z01). The location of the S753Y mutation in domain III of IF2 is shown as yellow spheres. Red spheres indicate the location of the Cy5 fluorophore appended to cysteine 810 in domain IV of IF2, and green spheres indicate the location of the Cy3 fluorophore appended to the 4-thiouridine at position 8 of fMet-tRNA^{fMet}.

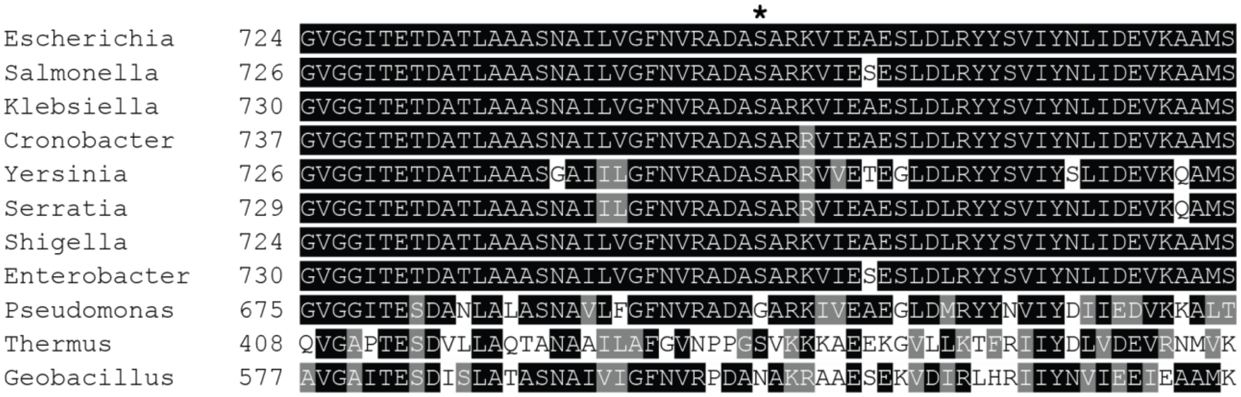

Supplementary Figure 2: Multiple sequence alignment of IF2. Multiple bacterial IF2 amino acid sequences were aligned using the T-coffee method[1] and shaded with BOXSHADE. Identical amino acids are shaded black and conservative substitutions are shaded gray. The asterisk indicates the serine in *E. coli* IF2 (S753) homologous to *S. typhimurium* IF2 S755.

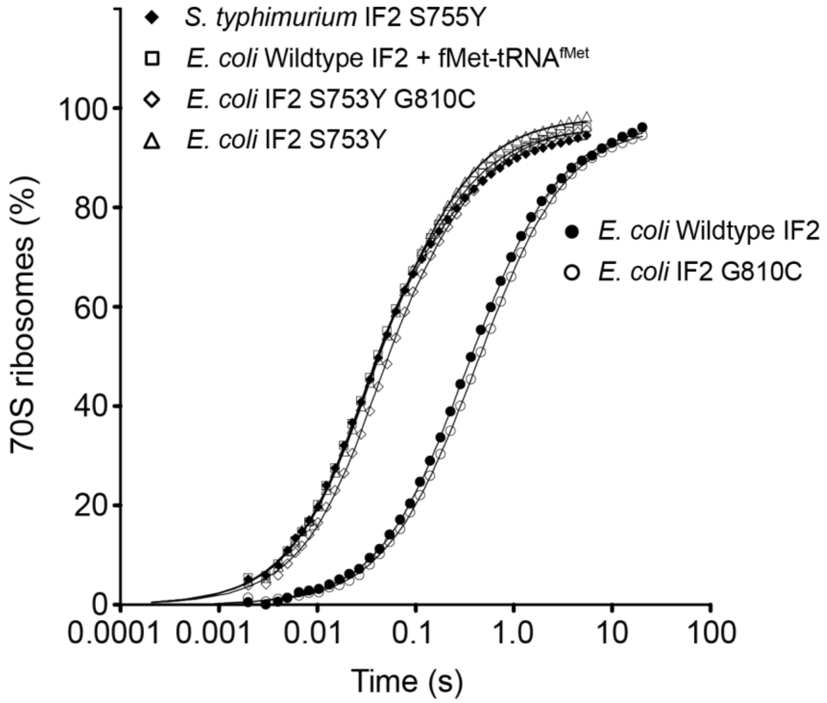

Supplementary Figure 3: Ensemble rapid kinetic studies of 50S subunit joining. The ensemble kinetics of 70S IC formation was determined after rapid mixing of 50S subunits and pseudo 30S ICs lacking fMet-$tRNA^{fMet}$, but carrying IF1 and the IF2 variants indicated. Rapid mixing of 50S subunits to 30S ICs assembled with *E. coli* wtIF2 and fMet-$tRNA^{fMet}$ was performed as a positive control.

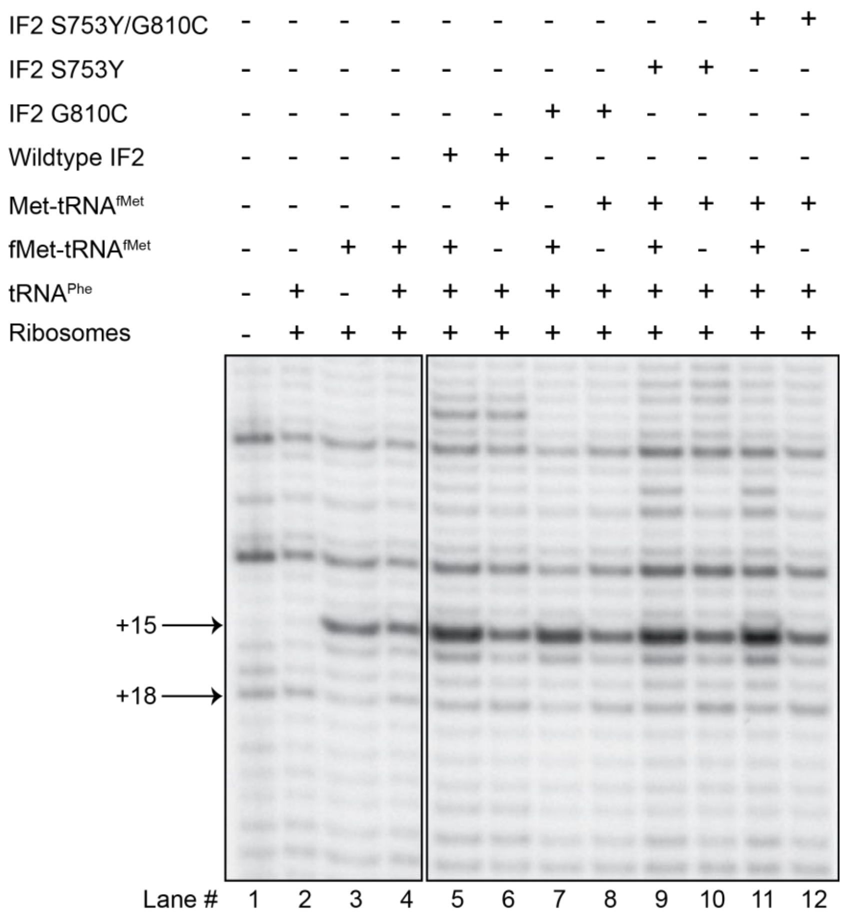

Supplementary Figure 4: Primer-extension inhibition, or "toeprinting", analyses of 30S ICs assembled on mRNApri-ext. The +15 band indicates that 30S subunits contain either Met-$tRNA^{fMet}$ or fMet-$tRNA^{fMet}$ in the P site and the +18 band indicates that 30S subunits have $tRNA^{Phe}$ in the P site. Reactions were performed in the absence (lanes 1-4) or the presence of the indicated IF2 variants (lanes 5-12). Lane 1 is a control reaction lacking ribosomes. Reactions performed in the absence of IF2 contained either $tRNA^{Phe}$ (lane 2), fMet-$tRNA^{fMet}$ (lane 3) or equimolar amounts of $tRNA^{Phe}$ and fMet-$tRNA^{fMet}$ (lane 4). Reactions performed in the presence of the indicated IF2 variant contained either equimolar amounts of $tRNA^{Phe}$ and fMet-$tRNA^{fMet}$ (lanes 5,7,9, and 11) or equimolar amounts of $tRNA^{Phe}$ and Met-$tRNA^{fMet}$ (lanes 6, 8, 10 and 12). All lanes were derived from the same 9% denaturing polyacrylamide gel.

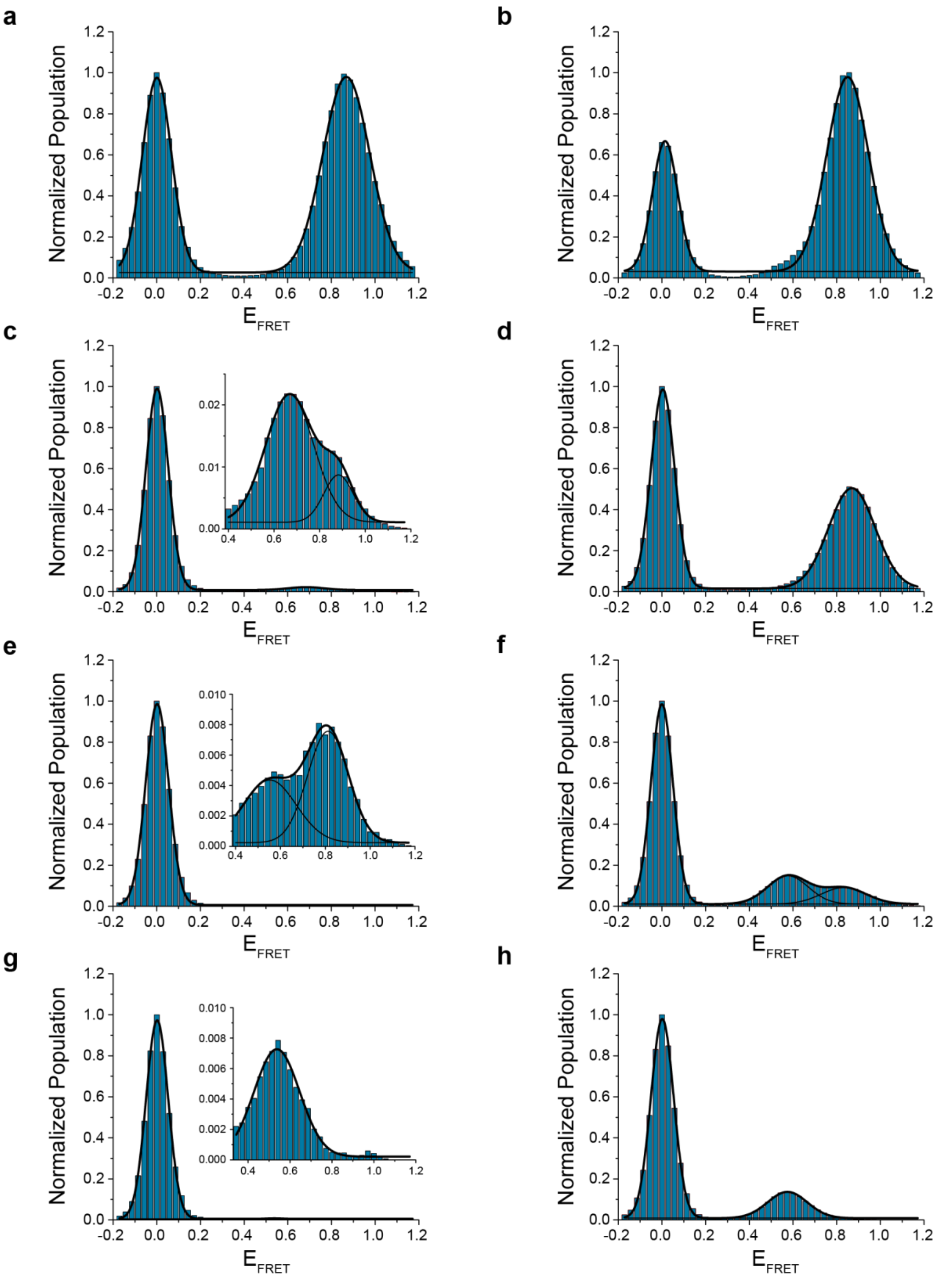
a
b
c
d
e
f
g
h
Normalized Population
E FRET

Supplementary Figure 5: Normalized one-dimensional $\boldsymbol{E_{\mathrm{FRET}}}$ histograms. Normalized one-dimensional E_{FRET} histograms for smFRET experiments reporting on the interaction of: (a) wtIF2[Cy5]$_{\mathrm{dIV}}$(GTP) with 30S IC$_{\mathrm{wT}}$, (b) mutIF2[Cy5]$_{\mathrm{dIV}}$(GTP) with 30S IC$_{\mathrm{mT}}$, (c) wtIF2[Cy5]$_{\mathrm{dIV}}$(GDP) with 30S IC$_{\mathrm{wD}}$, (d) mutIF2[Cy5]$_{\mathrm{dIV}}$(GDP) with 30S IC$_{\mathrm{mD}}$, (e) wtIF2[Cy5]$_{\mathrm{dIV}}$ with 30S IC$_{\mathrm{wT,Met}}$, (f) mutIF2[Cy5]$_{\mathrm{dIV}}$ with 30S IC$_{\mathrm{mT,Met}}$, (g) wtIF2[Cy5]$_{\mathrm{dIV}}$ with 30S IC$_{\mathrm{wT,OH}}$, and (h) mutIF2[Cy5]$_{\mathrm{dIV}}$ with 30S IC$_{\mathrm{mT,OH}}$. For the purposes of constructing the plots presented here, E_{FRET} histograms were generated using all of the data points prior to Cy5 photobleaching from the entire collection of raw E_{FRET} trajectories obtained from three independent replicates of each experiment. The E_{FRET} range from –0.2 to 1.2 was separated into 50 equally spaced bins and the histograms were normalized to the most populated bin. The resulting E_{FRET} histograms were fitted with multiple Gaussian distribution functions (black lines) using Origin 8.0 (Origin Lab Corp.). The insets in panels c, e, and g highlight the relatively small populations of E_{FRET} values corresponding to the non-zero FRET states that are observed in the larger histograms of the full populations of E_{FRET} values corresponding to all of the FRET states that are shown in the main panels c, e, and g. To generate the histograms shown in the insets, the small populations of E_{FRET} values corresponding to the non-zero FRET states were plotted independently of the relatively larger populations of E_{FRET} values corresponding to the zero FRET states. For the purposes of calculating the non-zero E_{FRET} values reported in the main text of the article and in Supplementary Table 1, Gaussian distribution functions were fit to the three E_{FRET} histograms corresponding to the three independent replicates of each experiment and the mean non-zero E_{FRET} values and standard deviations of the mean non-zero E_{FRET} values for each experiment were determined from the three fits. To evaluate the statistical significance of the differences between the mean non-zero E_{FRET} values that are reported in the main text of the article, paired *t* tests were carried out using Origin 8.0.

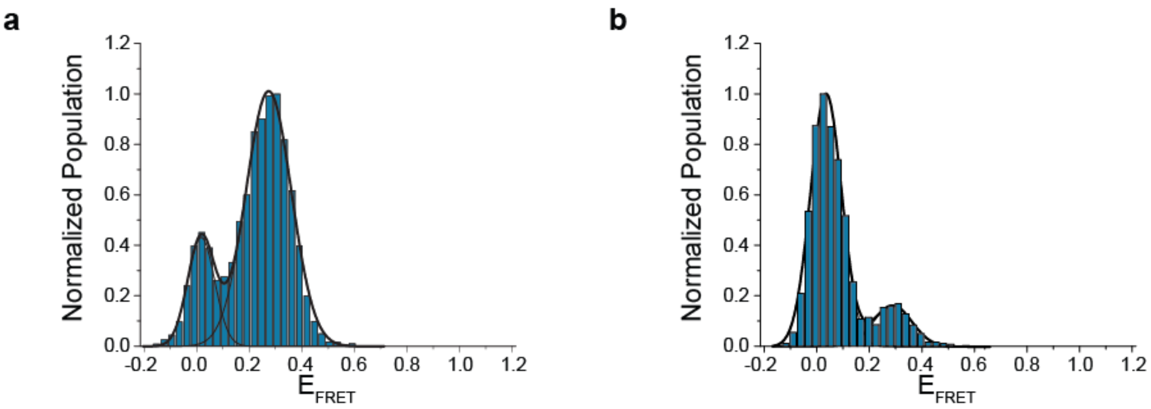

Supplementary Figure 6: Normalized one-dimensional E_{FRET} histograms. Normalized one-dimensional E_{FRET} histograms for smFRET experiments reporting on the interaction of: (a) wtIF2[Cy5]$_{dIII}$(GTP) with 30S IC$_{wT}$ and (b) wtIF2[Cy5]$_{dIII}$(GDP) with 30S IC$_{wD}$. For the purposes of constructing the plots presented here, E_{FRET} histograms were generated as described in Supplementary Figure 5, with the exception that the raw E_{FRET} trajectories were obtained from a single run of each experiment. For the purposes of calculating the non-zero E_{FRET} values reported in the main text of the article, Gaussian distribution functions were fit to the E_{FRET} histogram corresponding to each experiment and the mean non-zero E_{FRET} value was determined from the fit.

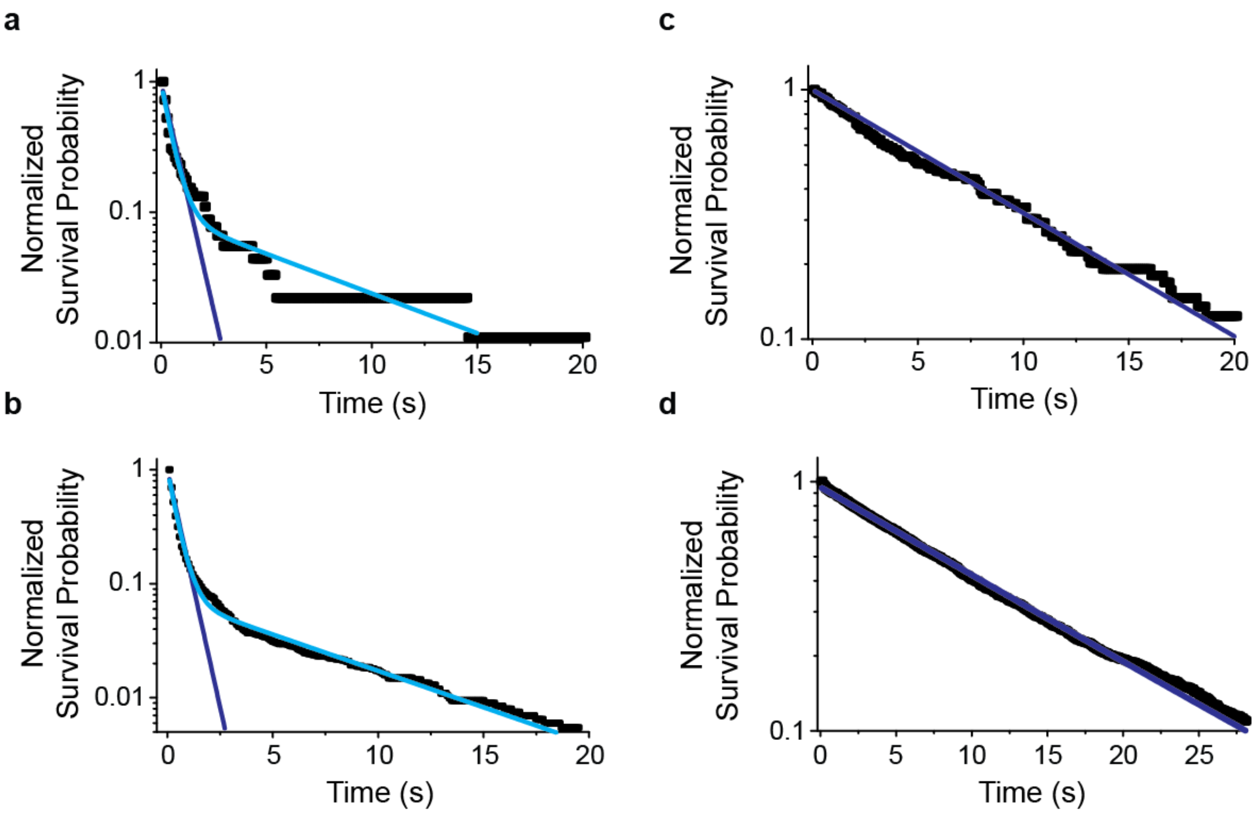

Supplementary Figure 7: Normalized survival probability plots of the wtIF2-bound state of the 30S IC. The entire collection of E_{FRET} trajectories obtained from smFRET experiments investigating the interaction of (a) wtIF2[Cy5]$_{\mathrm{dIII}}$(GDP) with 30S IC$_{\mathrm{wD}}$, (b) wtIF2[Cy5]$_{\mathrm{dIV}}$(GDP) with 30S IC$_{\mathrm{wD}}$, (c) wtIF2[Cy5]$_{\mathrm{dIII}}$(GTP) with 30S IC$_{\mathrm{wT}}$, (d) wtIF2[Cy5]$_{\mathrm{dIV}}$(GTP) with 30S IC$_{\mathrm{wT}}$, were first idealized to a hidden Markov model (HMM) using the vbFRET software package[2]. To separate dwells in the zero FRET-, wtIF2-free state of the 30S IC (hereafter referred to as the wtIF2-free state) and the non-zero FRET-, wtIF2-bound state of the 30S IC (hereafter referred to as the wtIF2-bound state), a threshold of E_{FRET} = 0.2 was applied to each of the idealized E_{FRET} trajectories and the dwell times in the wtIF2-bound state prior to transitioning into the wtIF2-free state were used to construct normalized survival probability plots[3] using OriginPro 8 (Origin Lab Corp.). To obtain the fractional occupancies and survival times of the wtIF2-bound state, the normalized survival probability plots were fit with a single-exponential decay function of the form

y = F*exp(–x/τ) (purple line), where F is the fractional occupancy, equal to 100 % in the single-exponential decay case, and τ is the survival time of the wtIF2-bound state. If necessary, the normalized survival probability plots were fit with a double-exponential decay function of the form y = F_1*exp(–x/τ_1)+(1–F_1)*exp(–x/τ_2) (light blue line), where F_1 and τ_1 are the fractional occupancy and survival time, respectively, of the first sub-population of the wtIF2-bound state and (1–F_1) and τ_2 are the fractional occupancy and survival time, respectively, of the second sub-population of the wtIF2-bound state. The normalized survival probability plot of the wtIF2[Cy5]$_{dIII}$(GDP)-bound state is best described by a double-exponential decay in which the minor sub-population exhibits a fractional occupancy of 9.7% and a survival time longer than the that of the fluorophores prior to photobleaching (~7.1 s in these experiments) and the major sub-population exhibits a fractional occupancy of 90.3% and a survival time of 0.46 s. This is in excellent agreement with the normalized survival probability plot of the wtIF2[Cy5]$_{dIV}$(GDP)-bound state, which is best described by a double-exponential decay in which the minor sub-population exhibits a fractional occupancy of 7.5% and a survival time longer than that of the fluorophores prior to photobleaching (~6.8 s in these experiments), and the major sub-population exhibits a fractional occupancy of 92.5% and a survival time of 0.42 s. In contrast, the survival probability plots of the wtIF2[Cy5]$_{dIII}$(GTP)-bound state and the wtIF2[Cy5]$_{dIV}$(GTP)-bound state are both best described by single-exponential decays with survival times longer than the those of the fluorophores prior to photobleaching (~8.8 s and ~11.8 s, respectively, in these experiments). Collectively, these data demonstrate that the wtIF2(GDP)-bound state observed using either wtIF2[Cy5]$_{dIII}$ or wtIF2[Cy5]$_{dIV}$ consists of two sub-populations, and that the kinetic properties of the two sub-populations corresponding to wtIF2[Cy5]$_{dIII}$ are indistinguishable from the kinetic properties of the two sub-populations corresponding to wtIF2[Cy5]$_{dIV}$.

Supplementary Tables

Supplementary Table 1: The non-zero mean E_{FRET} values, estimated distances between our fluorophore labeling positions, and sub-population occupancies of 30S IC-bound IF2.

30S IC	IF2	Nucleotide	E_{FRET}[a]	Distance (Å)[b]	Occupancy (%)[c]
**30S IC$_{wT}$**	wtIF2	GTP	0.87 ± 0.02	~40	100
**30S IC$_{mT}$**	mutIF2	GTP	0.85 ± 0.01	~41	100
**30S IC$_{wD}$**	wtIF2	GDP	0.67 ± 0.01	~49	82 ± 1.5
			0.89 ± 0.01	~39	18 ± 1.5
**30S IC$_{mD}$**	mutIF2	GDP	0.86 ± 0.03	~41	100
**30S IC$_{wT,Met}$**	wtIF2	GTP	0.55 ± 0.01	~53	44 ± 12
			0.81 ± 0.01	~43	56 ± 12
**30S IC$_{mT,Met}$**	mutIF2	GTP	0.57 ± 0.02	~52	58 ± 6
			0.83 ± 0.04	~42	42 ± 6
**30S IC$_{wT,OH}$**	wtIF2	GTP	0.53 ± 0.02	~54	100
**30S IC$_{mT,OH}$**	mutIF2	GTP	0.57 ± 0.01	~52	100

[a] Non-zero E_{FRET} values were determined from three independently collected datasets (mean ± SD) and represent the center of the Gaussian function fitted to the E_{FRET} histograms.

[b] The estimated distances between our fluorophore labeling positions were calculated using the equation $E_{FRET} = 1 / (1 + (R/R_0)^6)$ and assuming an R_0 value of 55 Å for the Cy3-Cy5 FRET pair[4].

[c] The sub-population occupancies were determined from three independently collected datasets (mean ± SD) and represent the normalized area of the Gaussian functions that were fitted to the E_{FRET} histograms.

Supplementary Table 2: The estimated occupancies of 30S IC-bound IF2 in our previously published ensemble rapid kinetic studies of 50S subunit joining[5].

Complex	IF2	Nucleotide	Occupancy (%)[a]
**30S IC_{wT}**	wtIF2	GTP	93.5
**30S IC_{mT}**	mutIF2	GTP	98
**30S IC_{wD}**	wtIF2	GDP	43
**30S IC_{mD}**	mutIF2	GDP	78
**30S $IC_{wT,Met}$**	wtIF2	GTP	19
**30S $IC_{mT,Met}$**	mutIF2	GTP	73
**30S $IC_{wT,OH}$**	wtIF2	GTP	9
**30S $IC_{mT,OH}$**	mutIF2	GTP	67

[a] Occupancy (%) = ([IF2•30S] / [30S]) $\times$ 100. [IF2•30S] was calculated by applying the quadratic binding equation using the equilibrium dissociation constants reported in Table 1 and the concentrations of IF2 and 30S subunits used in our previous ensemble rapid kinetic studies of subunit joining (0.6 μM and 0.32 μM, respectively)[5].

References

1. Notredame, C., Higgins, D. G. & Heringa, J. T-Coffee: A novel method for fast and accurate multiple sequence alignment. *J Mol Biol* **302,** 205–217 (2000).
2. Bronson, J. E., Fei, J., Hofman, J. M., Gonzalez, R. L. & Wiggins, C. H. Learning rates and states from biophysical time series: a Bayesian approach to model selection and single-molecule FRET data. *Biophys J* **97,** 3196–3205 (2009).
3. MacDougall, D. D. & Gonzalez, R. L. Translation initiation factor 3 regulates switching between different modes of ribosomal subunit joining. *J Mol Biol* **427,** 1801–1818 (2015).
4. Murphy, M. C., Rasnik, I., Cheng, W., Lohman, T. M. & Ha, T. Probing single-stranded DNA conformational flexibility using fluorescence spectroscopy. *Biophys J* **86,** 2530–2537 (2004).
5. Pavlov, M. Y., Zorzet, A., Andersson, D. I. & Ehrenberg, M. Activation of initiation factor 2 by ligands and mutations for rapid docking of ribosomal subunits. *EMBO J.* **30,** 289–301 (2011).
